# Characterization of the Striatal Extracellular Matrix in a Mouse Model of Parkinson’s Disease

**DOI:** 10.3390/antiox10071095

**Published:** 2021-07-08

**Authors:** Ana Freitas, Miguel Aroso, António Barros, Miriam Fernández, Eduardo Conde-Sousa, Marina Leite, Eva Daniela Carvalho, Cristina C Ribeiro, Rita Ferreira, Ana Paula Pêgo, Rui Vitorino, Maria Gomez-Lazaro

**Affiliations:** 1 i3S—Instituto de Investigação e Inovação em Saúde, Universidade do Porto, 4200-135 Porto, Portugal; anafreitas@ineb.up.pt (A.F.); miguel.aroso@i3s.up.pt (M.A.); econdesousa@ineb.up.pt (E.C.-S.); mleite@ipatimup.pt (M.L.); eva.carvalho@i3s.up.pt (E.D.C.); cribeiro@ineb.up.pt (C.C.R.); apego@i3s.up.pt (A.P.P.); 2 INEB—Instituto de Engenharia Biomédica, Universidade do Porto, 4200-135 Porto, Portugal; 3 FMUP—Faculdade de Medicina, Universidade do Porto, 4200-319 Porto, Portugal; asbarros@med.up.pt (A.B.); rvitorino@ua.pt (R.V.); 4 Department of Physiology and Cardiothoracic Surgery, Faculty of Medicine, University of Porto, 4200-319 Porto, Portugal; 5 Research Institute for Neurological Disabilities (IDINE), Medical School, University of Castilla-La Mancha, 02006 Albacete, Spain; miriam.fernandez@uclm.es; 6 IPATIMUP—Instituto de Patologia e Imunologia Molecular, Universidade do Porto, 4200-135 Porto, Portugal; 7 FEUP—Faculdade de Engenharia, Universidade do Porto, 4200-465 Porto, Portugal; 8 ISEP—Instituto Superior de Engenharia do Porto, Instituto Politécnico do Porto, Rua Dr. António Bernardino de Almeida 431, 4249-015 Porto, Portugal; 9 QOPNA & LAQV—Department of Chemistry, University of Aveiro, 3810-193 Aveiro, Portugal; ritaferreira@ua.pt; 10 ICBAS—Instituto Ciências Biomédicas Abel Salazar, Universidade do Porto, 4050-313 Porto, Portugal; 11 iBiMED—Department of Medical Sciences, University of Aveiro, 3810-193 Aveiro, Portugal

**Keywords:** extracellular matrix, Parkinson’s disease, oxidative stress, Raman spectroscopy

## Abstract

Parkinson’s disease’s etiology is unknown, although evidence suggests the involvement of oxidative modifications of intracellular components in disease pathobiology. Despite the known involvement of the extracellular matrix in physiology and disease, the influence of oxidative stress on the matrix has been neglected. The chemical modifications that might accumulate in matrix components due to their long half-live and the low amount of extracellular antioxidants could also contribute to the disease and explain ineffective cellular therapies. The enriched striatal extracellular matrix from a mouse model of Parkinson’s disease was characterized by Raman spectroscopy. We found a matrix fingerprint of increased oxalate content and oxidative modifications. To uncover the effects of these changes on brain cells, we morphologically characterized the primary microglia used to repopulate this matrix and further quantified the effects on cellular mechanical stress by an intracellular fluorescence resonance energy transfer (FRET)-mechanosensor using the U-2 OS cell line. Our data suggest changes in microglia survival and morphology, and a decrease in cytoskeletal tension in response to the modified matrix from both hemispheres of 6-hydroxydopamine (6-OHDA)-lesioned animals. Collectively, these data suggest that the extracellular matrix is modified, and underscore the need for its thorough investigation, which may reveal new ways to improve therapies or may even reveal new therapies.

## 1. Introduction

Parkinson’s disease is the second most prevalent neurodegenerative disease, affecting increasing numbers of people [1,2]. Currently, there is no cure for this disease, and its etiology remains unknown. The clinical motor symptoms associated with Parkinson’s disease result from the loss of specific dopaminergic neurons in the *Substantia nigra*, leading to the loss of striatal dopaminergic terminals. Increasing evidence points to the involvement of neuroinflammation and oxidative stress in the pathobiology of the disease [3]. Both contribute to increased levels of oxidative stress markers in the brain. Increased oxidative DNA damage, as well as lipid and protein oxidation of intracellular components, have been reported in samples from human patients and in animal models of the disease [4,5,6,7,8,9]. Strikingly, few studies have addressed the characterization of the specific modifications of the brain extracellular matrix (ECM) in this disease, although the ECM is increasingly recognized as an active player influencing cellular physiology [10,11,12,13]. The brain ECM has a unique composition with a high content in proteoglycans and glycoproteins and a low proportion of fibrous proteins [14]. Under oxidative stress, proteins and sugars are important targets for free radical modifications, resulting in specific post-translational modifications. These modifications can impair protein function by affecting enzymatic activity, access to binding sites, cellular recognition, protein aggregation, and proteolysis [15,16,17].

Proteomics studies using brain samples from patients and animal models have increased our knowledge on the pathobiology of the disease [18,19,20,21]. However, the exact contribution of ECM to the disease may have been overshadowed, because the brain ECM is a very challenging sample due to its low solubility [22]. Contributing to the low solubility of ECM are its highly cross-linked components [23], along with the high content and arrangement of high molecular weight components such as proteoglycans and glycosaminoglycans [24]. 

On the other hand, vibrational spectroscopy techniques do not require previous sample solubilization, representing an alternative technology for the chemical characterization of ECM. Nowadays, Raman spectroscopy is a widely used technology to study the biological changes occurring under various pathological conditions [25,26,27]. However, it has only recently been applied to the study of the ECM, mainly in the field of bioengineering. Kunstar and co-workers successfully used this methodology to monitor ECM deposition in bioengineered scaffolds by quantifying the Raman bands of collagen and sulfated glycosaminoglycans [28]. In addition, vibrational spectroscopy has also been used to detect changes induced by oxidative modifications in macromolecules or cells [29,30]. An example is the determination of protein carbonyl levels, recognized markers of oxidative stress [31]. Although several studies have used Raman spectroscopy to assess brain damage [25], to our knowledge, no research has been conducted on the examination of the striatal extracellular matrix in a mouse model of Parkinson’s disease using Raman spectroscopy. The fact that the brain ECM represents only 20% of the total tissue volume in the central nervous system [10], together with the complexity and chemical heterogeneity of this tissue, makes it challenging to study ECM modifications at a molecular level. However, the characterization of the striatal ECM may provide an important avenue for understanding the pathobiology of the disease and for discovering new targets for future clinical approaches.

In this regard, the present work aimed to characterize the striatal extracellular matrix in a mouse model of Parkinson’s disease. To this end, ECM components from striatal brain samples were first enriched and subsequently analyzed by Raman spectroscopy. A distinct chemical fingerprint was revealed in the matrix samples of the diseased animals, with increased oxidative modifications and the deposition of toxic aggregates. The effect of these modifications on the cellular response was next investigated by analyzing the activation of the microglia on the modified matrices, as well as the intracellular response to the modified matrix, using an intracellular actinin-sst-fluorescence resonance energy transfer (FRET) mechanosensor on US-O2 cells. We observed significant differences in the indicated mechanical strain of intracellular cytoskeletal fibers, demonstrating that cells respond differently to the decellularized matrices of the mouse model of the disease, suggesting that the mechanical changes resulting from a modified ECM could significantly affect cellular functions. Overall, these data demonstrate the presence of striatal ECM changes in a mouse model of Parkinson’s disease that could influence cell survival and physiology, possibly explaining the insufficient success of regenerative therapies based on cell transplantation in the diseased brain [32].

## 2. Materials and Methods

### 2.1. Unilateral 6-OHDA Lesion in the Substantia nigra

All of the experiments were performed according to National and European Union guidelines (EU Directive 2010/63/EU for animal experiments). Every effort was made to minimize animal discomfort and the total number of animals used. Procedures using animals were approved by the i3S Ethics Committee (CEA, i3S) and the Portuguese Veterinary Authorities (DGAV, license 15734/2018-08-29). Animals were kept in an enriched housing environment with *ad libitum* feed and water supply. They were kept under a 12 h light/12 h dark cycle in the i3S animal facility with controlled ambient temperature and humidity.

We used 34 male C57BL/6 mice aged 20–25 weeks weighing between 28–32 g. The animals were divided into three groups, as follows: Group 1, the Parkinson’s disease mice (PD), which received the stereotactic injection of 6-hydroxydopamine (6-OHDA; *n* = 11); Group 2, the saline/sham mice (SAL), in which the injection included a sterile saline solution (*n* = 10); and Group 3, the control mice (CTRL), in which no surgical procedure was performed (*n* = 13). The assignment of animals to each group was randomized and double-blinded.

Thirty minutes before the surgical procedure, the animals were injected intraperitoneally with the analgesic buprenorphine (0.08 mg/kg; Bupaq, richter-pharma, Wels, Austria) and the norepinephrine and serotonin reuptake inhibitor desipramine hydrochloride (Merck and Co., Inc., Kenilworth, NJ, USA; 0.01 mL/g) dissolved in 0.9% **w**/**v** sodium chloride (B. Braun, Hessen, Germany). Throughout the procedure, the eyes of the mice were protected with an ophthalmic gel to prevent dehydration (Siccafluid, Théa Laboratories, Clermont-Ferrand, France). Surgery was performed under volatile isoflurane anesthesia with body temperature control, and the animals were placed in a Kopf stereotaxic frame. The unilateral brain coordinates used for the *Substantia nigra* injection were as follows: A/P = −3.0 mm, M/L = −1.5 mm, and D/V = −4.6 mm. Because of the age and weight of the animals, these coordinates were previously established by injecting blue ink (methylene blue) into the brain using the same procedure. For the Parkinson’s disease mouse model (Group 1), 2 μL of 6-OHDA (5 µg/µL; H116, Merck, Darmstadt, Germany) was injected at a rate of 0.3 µL/min using a Hamilton Neuros syringe with a 33G needle (Hamilton, Bonaduz, GR, Switzerland). After injection, the skin was sutured and sterile 0.9% **w**/**v** sodium chloride (B. Braun) was administered subcutaneously to hydrate the mice. The same procedure was performed in sham animals (Group 2), where 2 µL of sterile 0.9% sodium chloride (B. Braun) was administered into the *Substantia nigra*. Immediately after surgery, the animals were placed on a heating pad for 30 min to recover. For the first days after surgery, the mice received subcutaneous injections of Duphalyte solution (Zoetis, NJ, USA) upon abnormal weight loss, supplemental food (*ad libitum* diet-recovery gel (ClearH_2_O, Portland, OR, USA)), moist regular food pellets, and sunflower seeds. The animals were monitored daily, and additional supplemental feeds were administered orally when needed (Anima strath, Bio-strath, Zurich, Switzerland). Humane endpoints were performed when weight loss exceeded 20%. Fourteen days after surgery, lesion severity was assessed by quantifying the apomorphine-induced rotations. The animals were transferred to new cages and were allowed to habituate for 15 min before the subcutaneous injection of apomorphine (apomorphine solution (0.1 mg/Kg (R)-(-) apomorphine hydrochloride; Biogen Científica, Madrid, Spain), 0.2 mg/mL L-ascorbic acid (Biogen Científica, Madrid, Spain), in 0.9% *w*/*v* sterile sodium chloride (B. Braun)). Five minutes after injection, the number of rotations was recorded for 30 min [33].

The animals were euthanized 21 days after surgery under carbon dioxide aspiration. The brains were immediately extracted, cut into 250 µm thick coronal sections (Leica VT1000S, Leica Biosystems, Wetzlar, Germany), and stored at −80 °C for further processing. Loss of the dopamine neuron was confirmed by immunostaining the striatal sections with an antibody against tyrosine hydroxylase (ab112, Abcam, Cambridge, UK; Figure S1).

### 2.2. Decellularization of Striatal Brain Slices

We applied a decellularization procedure to the striatal slices to enrich the samples on the ECM components. To this end, sections were incubated at room temperature, under orbital agitation (150 rpm) for 72 h, with 0.1% sodium dodecyl sulfate solution (SDS; total volume 160 µL) supplemented with penicillin-streptomycin solution (1% (*v*/*v*), L0022, Biowest, Nuaillé, France), and gentamicin (50 µg/mL, G1272, Merck, Darmstadt, Germany). The SDS solution was replaced every 24 h. After this period, the samples were washed with 1× PBS (137 mM NaCl, 2.7 mM KCl, 10 mM Na_2_HPO_4_, 1.8 mM KH_2_PO_4_, pH 7.4, all reagents from Merck) and centrifuged at 20,000× *g* for 3 min. This step was repeated three times to remove any residual SDS detergent. All of the samples were stored at −80 °C until further use.

To assess the cell content removal, samples were incubated with 1 µg/mL DAPI (4′,6-diamidino-2-phenylindole; D9542, Merck) for 1 h at RT for nuclear staining. Images were acquired with a widefield microscope (Axiovert 200M, Carl Zeiss AG, Oberkochen, Germany) using an EC Plan-Neofluar 10× NA 0.30 Ph1 objective. Additionally, we assessed the content of the intracellular protein actin in the decellularized matrices using Western blot.

Estimation of the ECM preservation was performed by Alcian Blue staining to detect glycosaminoglycans in the decellularized matrices, using previously described protocols [34,35,36] with slight modifications. Briefly, the samples were thawed and placed in type II water for 2–3 min. After this time, the water was removed, and all of the samples (decellularized ECMs (dECMs) and non-decellularized ECMs (ECMs)) were incubated with 3% (*v*/*v*) glacial acetic acid (Merck, Darmstadt, Germany) for 3 min at RT. After removing the glacial acetic acid, 1% (*w*/*v*) Alcian Blue (Alcian Blue 8GX, A9186, Merck, Darmstadt, Germany) in 3% glacial acetic acid was added to the sections and kept for 1 h at RT. After two washes with type II water, the samples were mounted into microscope glass slides with a Mowiol 4-88 solution (5.4 M Mowiol 4-88, 67% *v*/*v* 1× PBS, 33% (*v*/*v*) glycerol ultrapure, mixed with *n*-propyl gallate (39.3 mM *n*-propyl gallate, 50%(*v*/*v*) glycerol, 50% (*v*/*v*) 1× PBS, all from Merck). Images were acquired using a widefield microscope (Axiovert 200M, Carl Zeiss AG) using an EC Plan-Neofluar 10× NA 0.30 Ph1 objective.

### 2.3. Protein Immunodetection on Striatal Slices

To detect the expression of tyrosine hydroxylase (TH), brain slices were placed in cold 4% paraformaldehyde (11473704, Fisher Scientific, MA, USA) for 30 min immediately after sectioning. The sections were then incubated with a solution of 0.2% Triton X-100 in PBS for 10 min at RT with gentle agitation. Two washes with 1× PBS were performed, followed by a blocking step with a blocking buffer (1% BSA, Merck; 10% fetal bovine serum (FBS), Biowest; 0.2% Triton X-100, Merck; dissolved in 1× PBS) for 2 h at RT. The slices were then incubated with 2 µg/mL of the primary anti-TH antibody (Abcam) at 4 °C overnight. After washes three times with 1× PBS (5 min each), the samples were incubated with 2 µg/mL of the secondary antibody anti-Rabbit-Alexa Fluor^®^ 647 (chicken anti-Rabbit A-21443, Thermo Fisher Scientific, MA, USA). After washing three times with 1× PBS (5 min each), the samples were incubated with DAPI (2 μg/mL, D9542, Merck, Darmstadt, Germany) for 1 h at RT. Finally, the samples were mounted on glass slides with a Mowiol 4-88 solution, after a final wash with 1× PBS.

To detect the ECM components in the decellularized brain sections, samples were thawed and then fixed with 4% paraformaldehyde for 20 min at RT. The sections were then incubated with 0.2% Triton X-100 in 1× PBS for 7 min at RT, followed by washing with 1× PBS (twice, 5 min each). After a 2 h incubation at RT with the blocking buffer, the decellularized sections were incubated with primary antibodies diluted in the same blocking solution. Depending on the antibodies used, the decellularized samples were incubated with either 4 μg/mL of anti-collagen IV antibody (ab6586, Abcam) or 4 μg/mL of anti-fibronectin antibody (ab2413, Abcam) for 38 h at 4 °C. After washing three times with 1× PBS (5 min each), the samples were incubated with 2 µg/mL of anti-Rabbit Alexa Fluor^®^ 647 secondary antibody (A-21443, Thermo Fisher Scientific) for 1 h at RT. The samples were mounted on glass slides with Mowiol 4-88 solution, after a final wash with 1× PBS.

The images were acquired with a laser scanning confocal microscope (Leica TCS SP5 II, Leica Microsystems), using a HCX PL APO CS 10x 0.40 NA dry UV objective, and lasers with wavelengths of 405 and 633 nm for DAPI and Alexa Fluor^®^ 647 signal acquisition, respectively.

### 2.4. Immunoblotting Assays

The human neuroblastoma SH-SY5Y cell line (ATCC, CRL-2266) was used as a control for the cell content. This catecholaminergic cell line has been widely used as a model for Parkinson’s disease [37]. They were cultured in T75 flasks with DMEM-F12 medium (Corning Life sciences, NY, USA) supplemented with 10% (*v*/*v*) fetal bovine serum (Biowest) and 1% (*v*/*v*) penicillin-streptomycin solution (L0022, Biowest) under a standard incubation at 37 °C and 5% CO_2_. The medium was changed every two days. When confluence of approximately 70–80% was reached, the cells were scraped and collected using ice-cold sterile 1× PBS and centrifuged at 20,000× *g* for 3 min at 4 °C. The pellet was frozen in liquid nitrogen and stored at −80 °C until further use.

The ECMs, dECMs, and SH-SY5Y cell pellets were solubilized with a guanidine hydrochloride buffer (4 M Gdn-HCl; 50 mM sodium acetate, pH 5.8, and 25 mM EDTA (Merck) supplemented with phosphatase and protease inhibitors (biotools, B15001, New Taipei City, Taiwan, and bimake.com, B14001, TX, USA). The samples were resuspended in 10 volumes of Gdn-HCl buffer and were incubated under rotation (550 rpm) for 72 h at RT. Every 24 h, the samples were pipetted up and down several times. Then, the samples were briefly vortexed for 1 min and were sonicated for approximately 7 min. This step was repeated 2–3 times until the solution took on an opaque color. After a final centrifugation step (20,000× *g* for 15 min at 4 °C), the supernatant was collected into a new tube.

The samples were then precipitated by adding six volumes of cold ethanol (99.9%) and were incubated overnight at −20 °C, followed by a centrifugation step at 14,000× *g* for 45 min at 4 °C. The pellets were air dried and later resuspended in a total volume of 80 μL in a rehydration buffer (7 M urea, 2% CHAPS, all from Merck). The total precipitated protein content was quantified using the Bio-Rad RC/DC kit (Protein assay, #5000121, Bio-Rad Laboratories, Inc., CA, USA) according to the manufacturer’s instructions.

Forty micrograms of extracted protein from each condition (ECM, dECM, and the cell line SH-SY5Y) were resolved into a 7.5% SDS-PAGE followed by a 2-h wet transfer. The nitrocellulose membrane was placed in a blocking solution (BSA 5% in 1× TBS) for 1 h at RT. They were then incubated overnight at 4 °C with gentle rotation with monoclonal antibodies, as follows: anti-actin (0.5 μg/mL, 5% skimmed milk in 1× TBS/0.1% Tween^®^ 20, A3853, Merck, Darmstadt, Germany) and anti-brevican (2 μg/mL, 5% skimmed milk in 1× TBS/0.1% Tween^®^ 20, HPA007865, Merck). The following day, a 1-h incubation was performed at RT with the secondary antibodies (3 μg/mL of anti-mouse, NA931and anti-rabbit antibody, NA934, respectively; Amersham GE, Cytiva, MA, USA). Protein expression was detected by enhanced chemiluminescence (ECL) according to the manufacturer’s instructions (Cytiva).

### 2.5. Confocal Raman Microspectroscopy

The samples were mounted on CaF_2_ coverslips (Crystran Ltd., Dorset, UK) and dried for 24 h at RT (unlabeled samples). For the analysis of the dinitrophenyl (DNP) derivatives, the samples were incubated with 10 mM DNPH (2,4-dinitrophenylhydrazine, D199303) in 10% trifluoroacetic acid (TFA; both from Merck) for 15 min. After washing three times with type II water, they were placed on CaF_2_ coverslips and dried for 24 h at RT (DNPH-labelled samples). Confocal Raman microspectroscopy analyses were performed using a LabRAM HR 800 confocal Raman microscope system (Horiba Jobin Yvon, Tokyo, Japan), which included a spectrometer and a fully integrated Olympus BX41 confocal microscope (Olympus Corp., Tokyo, Japan). Raman spectra were acquired using a 632.4 nm laser diode as the excitation source, covering two spectral regions depending on the purpose of the analysis. The range from 0 to 3800 cm^−1^ was chosen for the characterization of the ECM-specific fingerprint (unlabeled samples), while the range of 400–1800 cm^−1^ was chosen for the specific analysis of DNPH-labeled carbonyls (DNPH-labelled samples). All of the spectra were acquired with an Olympus MPlan 100× objective (N.A. 0.90, Olympus) and a pinhole aperture of 50 µm using the LabSpec 5 software (version 5.25.15, Horiba Jobin Yvon). The spectra were recorded from randomly selected points in the striatal samples, with an integration time of 120 s and two accumulations. A grating dispersed the scattered light at 1800 lines/mm (Jobin Yvon) with a spectral resolution of 0.21 cm^−1^/point. The spectra were analyzed using the R language environment software (version 3.5). Briefly, the workflow began with the inspection and removal of error spectral, after which the spectra were baseline corrected and normalized. Multivariate analysis was performed using Orthogonal Projections to Latent Structures Discriminant Analysis (OPLS-DA) to decipher the characteristic Raman features. Peaks relevant to the changes (VIP > 1) were further analyzed using the IBM^®^ SPSS^®^ Statistics software (version 25, IBM^®^ Corporation, Armonk, NY, USA) by a one-way ANOVA or Welch’s ANOVA test followed by Bonferroni or Games-Howell post hoc test, respectively.

### 2.6. Assessment of the Intracellular Mechanical Stress

For the assessment of the intracellular mechanical stress of the cells growing in the decellularized matrices, the U-2 OS (ATCC^®^ HTB-96™) cell line was used together with the following DNA plasmids: actinin-M-sstFRET (Kindly provided by Dr. Fanjie Meng, Center for Single Molecule Biophysics, Department of Physiology and Biophysics, The State University of New York at Buffalo, USA) [38], Cerulean-N1 (#54742, Addgene), and mVenus C1 (#27794, Addgene).

For the experimental setup, U-2 OS cells were seeded on six-well plates (1 × 10^5^ cells/well) and maintained in culture overnight under standard CO_2_ (5%) environment at 37 °C. Lipofectamine transfection was performed according to the manufacturer’s guidelines (Lipofectamine 3000, Invitrogen Thermo Fisher Scientific). We transfected a total of 2 µg of DNA (sst-FRET actinin M, Venus and Cerulean, respectively) in a low-serum medium (OPTI-MEM, Gibco Thermo Fisher Scientific), and the cells were incubated for 5 h. After this incubation period, the medium was changed to a regular medium (DMEM-F12 medium (Corning Life sciences) supplemented with 10% (*v*/*v*) fetal bovine serum (Biowest) and 1% penicillin-streptomycin solution (L0022, Biowest). Twenty-four hours later, the cells were trypsinized and used to repopulate the dECMs. Prior to this cell seeding, brain dECMs were placed on µ-Slide eight-well glass bottom chamber slides (ibidi, Gräfelfing, Germany), and gently dried under sterile conditions for 30 min, followed by the addition of regular media.

Twenty-four hours after seeding the cells into the dECMs, the fluorescence resonance energy transfer (FRET) signals were acquired with a laser scanning confocal microscope (Leica TCS SP5, Leica microsystems), using the 458 and 514 nm laser lines and the 63 × HCX PL APO CS oil immersion objective with 1.40 NA. The cells were maintained at 37 °C and 5% CO_2_ throughout the entire scanning procedure.

The sensitized emission method was used to evaluate the FRET efficiencies. Fluorescent signals from the donor and acceptor were obtained in a sequential acquisition line by line. The cell expressing only donor (Cerulean) and acceptor (Venus) were used as controls. These controls were selected to obtain the calibration coefficients to correct the crosstalk for excitation and emission. The 458 nm laser line was used for excitation, while the detection range for the donor and acceptor were 462–510 nm and 518–580 nm, respectively. The images were acquired and calibrated, and the FRET efficiencies were obtained following the FRET SE method of LAS AF software (version 2.6.0.7266, Leica microsystems). A ratiometric calculation was used to quantify the FRET efficiencies (FRET signal/donor signal) because the actinin-M-sstFRET plasmid has a fixed stoichiometry of 1:1 for the donor and acceptor.

### 2.7. Isolation of Primary Microglia

Microglia primary cultures were obtained from postnatal Wistar rat pups (P0-P2) following previously described protocols [39]. After decapitation, the brain cortex was dissected and digested with 0.0025% (*w*/*v*) trypsin-EDTA (Gibco 25200, Thermo Fisher Scientific) and 0.001 mg/mL DNAse I (Sigma D5025-15KU, Merck and Co., Inc., Kenilworth, NJ, USA) for 15 min at 37 °C. Enzymes were inhibited by the addition of DMEM Glutamax High Glucose (Thermo Fisher Scientific, MA, USA) supplemented with 10% (*v*/*v*) heat-inactivated FBS (Merck and Co., Inc., Kenilworth, NJ, USA) and 1% (*v*/*v*) penicillin/streptomycin (L0022, Biowest, Nuaillé, France). After centrifugation at 500× *g* for 10 min at RT, the cells were resuspended in DMEM (Thermo Fisher Scientific, MA, USA) supplemented with 10% (*v*/*v*) heat-inactivated FBS (Merck and Co., Inc., Kenilworth, NJ, USA) and 1% (*v*/*v*) penicillin/streptomycin (L0022, Biowest) and filtered through a 40 μm nylon cell strainer. The cells were seeded on flasks coated with 100 μg/mL poly-L-lysine (L 7240, Biochrom GmbH, Berlin, Germany) and were cultured at 37 °C and 5% CO_2_ conditions for ten days. To collect microglia, flasks with growing cells were shaken at 210 rpm at 37 °C for 2 h. The microglia were collected from the supernatant by further centrifugation at 200× *g* for 5 min. The purity of microglia cultures was found to be more than 90%.

### 2.8. Repopulation of the Decellularized Matrices

The decellularized brain slices were placed on μ-Slide 8 Well Glass Bottom chamber slides for cell imaging (ibidi, Gräfelfing, Germany) under sterile conditions and allowed to dry for 30 min in the flow hood. Primary microglia cells were then seeded at a density of 1.5 × 10^5^ cells/well in DMEM-F12 medium (Corning Life sciences, NY, USA) supplemented with 10% FBS (Biowest) and 1% (*v*/*v*) penicillin/streptomycin (L0022, Biowest), and were cultured under standard CO_2_ (5%) and 37 °C conditions. Forty-eight hours later, the cells were fixed with warm 4% paraformaldehyde (Merck) for 30 min at RT. After three washing steps with 1× PBS (5 min each), the samples were permeabilized by incubation with 0.2% Triton X-100 in 1× PBS for 7 min. Then, the samples were incubated with a blocking solution (1% BSA (*w*/*v*; Merck, Darmstadt, Germany), 10% (*v*/*v*) FBS (Biowest), and 0.02% (*v*/*v*) Tween^®^ 20 (Merck) in 1× PBS) at RT. The samples were then incubated with 2 μg/mL of anti-IBA-1 antibody (Wako Fujifilm chemicals, Osaka, Japan) for 38 h at 4 °C. After washing the primary antibody three times with 1× PBS, the samples were then incubated with 2 μg/mL of anti-Rabbit Alexa Fluor^®^ 647 antibody for 1 h at RT (Thermo Fisher Scientific, MA, USA) and counterstained for nuclei staining with 2 μg/mL DAPI (D9542, Merck). After a final wash with 1× PBS, the samples were mounted in glass coverslips with Mowiol 4-88 solution.

To assess the effect of a modified ECM on the microglia, 48 h after repopulation, the nuclei of IBA-1-positive cells were counted in seventeen random fields for three independent experiments.

### 2.9. Analysis of the Microglial Morphological Features

Images were acquired with a widefield microscope (Axiovert 200M, Carl Zeiss AG, Oberkochen, Germany) using a 40× LD Plan-Neofluar oil immersion objective 0.6 N.A., and appropriate filters for DAPI and Alexa Fluor^®^ 647. To quantify the morphological features of the microglia, images were processed using ImageJ [40,41] and Ilastik [42] software. Briefly, the rolling ball algorithm was first applied with a radius of 80 pixels to fix the uneven background. The resulting images were then segmented within Ilastik, an interactive machine learning framework for pixel segmentation. A second stage for segmentation refinement was then performed in ImageJ to split touching cells (by manually deleting a line with two pixels width) or to merge processes incorrectly separated from the corresponding cell bodies. For each resulting image/mask pair, measurements of area (µm^2^), perimeter (µm), major and minor axes, roundness, Feret’s diameter (µm), aspect ratio, solidity, and circularity were made. The definition of each feature is defined as the total number of pixels present in an object. The feature perimeter is defined as the length of the outer boundary of the cell. The major axis is defined as the longest line that can be drawn through the cell, while the minor axis is the longest line that can be drawn through the cell while remaining perpendicular to the major axis. The shape descriptor circularity is calculated using the following formulae:

(1) $$c=\frac{4\pi \times area}{{\left(perimeter\right)}^{2}}$$

In which *c* = 1 represents a perfect circle, while values closer to *c* = 0 (linear polygon) represent a more elongated shape. The Feret’s diameter feature is defined as the longest distance between any two points, also known as the maximum caliper. The aspect ratio feature is defined as the ratio between the distance in pixels of the major axis and the distance in pixels of the longest line drawn through the object while perpendicular to the major axis, while the roundness feature can be defined as the ratio of the area of an object to the area of a circle with the same convex perimeter by applying the following formulae:

$$r=\frac{4\pi \times area}{{\left(convex\;perimeter\right)}^{2}}$$

with *r* = 1 for a circular object and *r* < 1 for more elongated. Finally, the solidity feature, which measures the density of an object, is calculated with the following formulae:

(2) $$s=\frac{area}{convex\;area}$$

*s* values closer to 1 point to a solid object, while *s* values closer to 0 indicate that the object’s boundary is irregular.

When applied, statistical analysis was performed using the IBM^®^ SPSS^®^ Statistics software (version 25, IBM^®^ Corporation, Armonk, NY, USA). A Welch’s ANOVA test was followed by a Games-Howell post hoc test to identify which groups were different. Statistical significance was set at *p* ≤ 0.05 (## *p* ≤ 0.001, # *p* ≤ 0.05).

## 3. Results

### 3.1. Striatal Extracellular Matrix Enrichment

**Figure 1:**
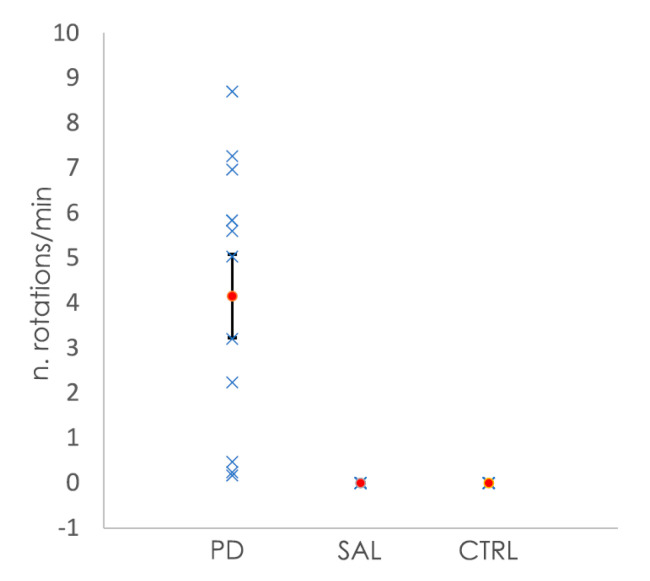
Representation of the number of rotations per minute shown by the mice after the injection of apomorphine. Animals subjected to the 6-OHDA lesion showed rotational behavior, while the control animals (CTRL) and animals injected with saline (SAL) did not show any rotation. In blue are the values of the number rotations per minute for each animal, while the orange dots represent the mean number of rotations per minute per group. Bars for the SEM are shown in the graph.

All of the animals subjected to the 6-OHDA lesion showed rotational behavior after apomorphine injection, whereas the saline-injected and control animals showed no rotations after the injection (Figure 1). The quantification of the number of rotations per minute allowed for the classification of the severity of the clinical phenotype, from mild to severe [33]. In the case of the animals from the 6-OHDA-lesioned group, those with mild changes had a rotation value (mean ± SEM) of 0.78 ± 0.49 rotations/min (four animals, with values between 0.2 to 2.2 rotations/min). Moderate changes were represented by a mean ± SEM of 4.92 ± 0.60 rotations/min (four animals, with values between 3.2 and 5.8 rotations/min), while animals with severe changes had a mean ± SEM of 7.64 ± 0.53 (three animals, values between 7.0 to 8.7 rotations/min).

**Figure 2:**
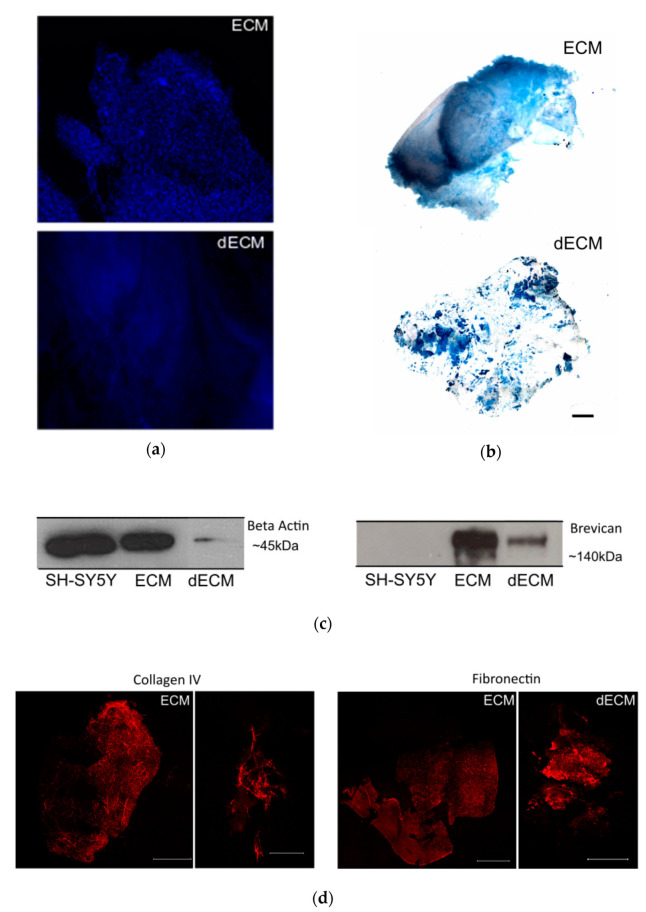
Decellularization of the striatal sections. (**a**) Non-decellularized (ECM) and decellularized (dECM) sections (250 µm thickness) from the striatum region of a C57/BL6 mouse brain stained with the nuclear dye DAPI (in blue) to assess the removal of cellular components; scale bar 100 µm. (**b**) The Alcian Blue staining of the ECM and dECM slices highlight the preservation of the glycosaminoglycans; scale bar 800 µm. (**c**) Detection of intracellular protein actin (left) and proteoglycanbrevican (right) by Western blot. It was possible to confirm the reduction of the cellular content and the preservation of ECM components on the dECM samples, respectively. Actin was present in high amounts in the solubilized non-decellularized ECM slices and in the lysate of the SH-SY5Y control cell line, whereas it was drastically reduced in the solubilized dECMs. (**d**) Immunostaining of collagen IV and fibronectin proteins in both ECM and dECM slices. The scale bar for ECM images is 1500 μm, while for the dECM collagen IV image it is 500 μm and for the dECM fibronectin image it is 1500 μm.

To characterize the striatal ECM, we previously enriched our samples for ECM components by a decellularization process, by incubating the striatal slices with the ionic detergent SDS (described in the Materials and Methods section). Macroscopically, the striatal brain slices changed from a previously white-dense appearance to white-translucent structures after the decellularization process (data not shown). The decellularized sections enriched with ECM components exhibited a significant reduction in cellular content, as shown by the loss of nuclear staining in the samples from the decellularized matrices (dECM) compared with the native (ECM; Figure 2a). In addition, the reduction in the intracellular content was illustrated by a marked decrease in the immunodetection of the intracellular protein actin in the samples from the decellularized matrices (Figure 2c).

To monitor the preservation of the extracellular matrix components after the decellularization process, we performed Alcian Blue staining. This cationic dye is commonly used for the staining of glycosaminoglycans [43], which are eminently present in the brain ECM [14]. From Figure 2b, it was confirmed that glycosaminoglycans were preserved after the decellularization process. To assess the presence of extracellular matrix proteins in the decellularized slices, we also performed immunostaining with specific antibodies against the ECM proteins collagen IV and fibronectin. As shown in Figure 2d, it was possible to detect both proteins after the decellularization process, highlighting the preservation of extracellular matrix components, similar to what was observed for the ECM protein brevican by Western blot (Figure 2b).

### 3.2. Characterization of the Striatal Extracellular Matrix by Raman Spectroscopy

#### 3.2.1. Raman Fingerprint of the Striatal Extracellular Matrix in a Mouse Model of Parkinson’s Disease

Immunohistochemistry is a commonly used technique for the analysis of tissue changes in disease. However, its application is limited by the availability of dyes or antibodies to detect or identify a particular component or chemical modification. Moreover, the selection of the specific label requires prior knowledge of the alteration under investigation. On the other hand, proteomics-based technologies can provide qualitative and quantitative information about tissue composition and chemical changes; however, they require a relatively large sample size and time-consuming processing protocols that are not ideal for every component of the brain extracellular matrix, given its complex composition and difficult solubilization [23]. In this regard, vibrational spectroscopy techniques have been proven to be powerful tools for determining the chemical fingerprint of specific tissues and conditions, with few or no prior sample preparation steps. In addition, they require a small amount of sample. In the present work, confocal Raman microspectroscopy was used to investigate the striatal extracellular matrix changes in a mouse model of Parkinson’s disease. Raman spectra of 2–10 spatial points were collected from each section of the decellularized matrices, with 1–3 sections per animal and a total of 9–12 animals per condition. Table 1 shows the peaks that appeared in the Raman spectra from these samples with their corresponding assignments.

**Figure 3:**
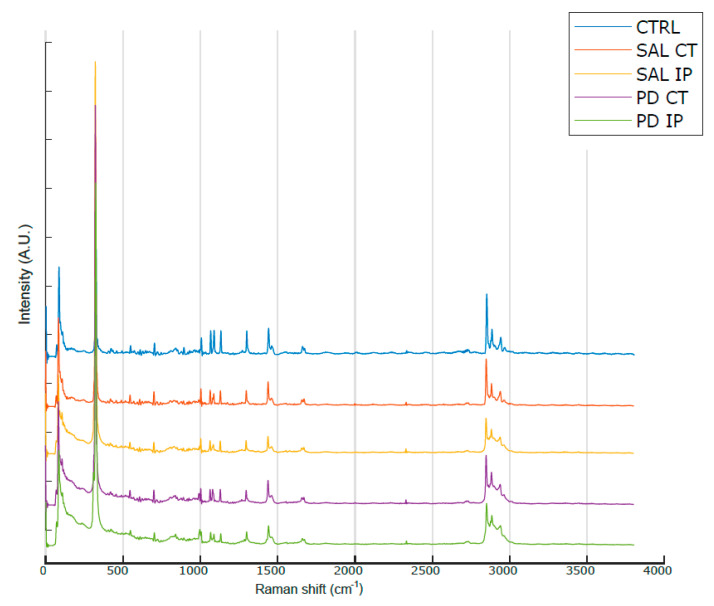
Average Raman spectra from the striatal decellularized matrices of the control mice and the contralateral and ipsilateral hemispheres of the saline- and 6-OHDA-lesioned mice between the range of 0 and 3800 cm^−1^. For representation purposes, Matlab Scheme 9. 4.0.813654 (R2018a). The MathWorks Inc., Natick, MA, USA, was used to obtain the average smoothed spectra with background subtraction for the striatal decellularized matrices from control mice (CTRL; total of 100 spectra in blue), saline contralateral hemisphere (SAL CT; total of 60 spectra in orange), saline ipsilateral hemisphere (SAL IP; total of 44 spectra in yellow), 6-OHDA-lesioned contralateral hemisphere (PD CT; total of 56 spectra in purple), and 6-OHDA-lesioned ipsilateral hemisphere (PD IP; total of 54 spectra in green). The spectra are offset along the intensity axis for clarity. Information on the assigned peaks can be found in Table 1.

Figure 3 shows the average Raman spectra, baseline-corrected and smoothed, from the decellularized matrices of the control, saline and 6-OHDA-lesioned mice (both contralateral and ipsilateral hemispheres) recorded in the range between 0 and 3800 cm^−1^. The most prominent peaks appeared in the Raman spectra within the intervals of 83–290 cm^−1^ and 2800–3000 cm^−1^, which correspond to the regions attributed to the bending of oxalates and symmetric and asymmetric stretching of CH_2_ and CH_3_, respectively. Interestingly, the latter region was also observed in the Raman spectra obtained from monkey’s *Substantia nigra* samples in a Parkinson’s disease model [25], although the complete tissue was used for the analysis.

#### 3.2.2. Alterations of the Striatal Extracellular Matrix in a Mouse Model of Parkinson’s Disease

**Figure 4:**
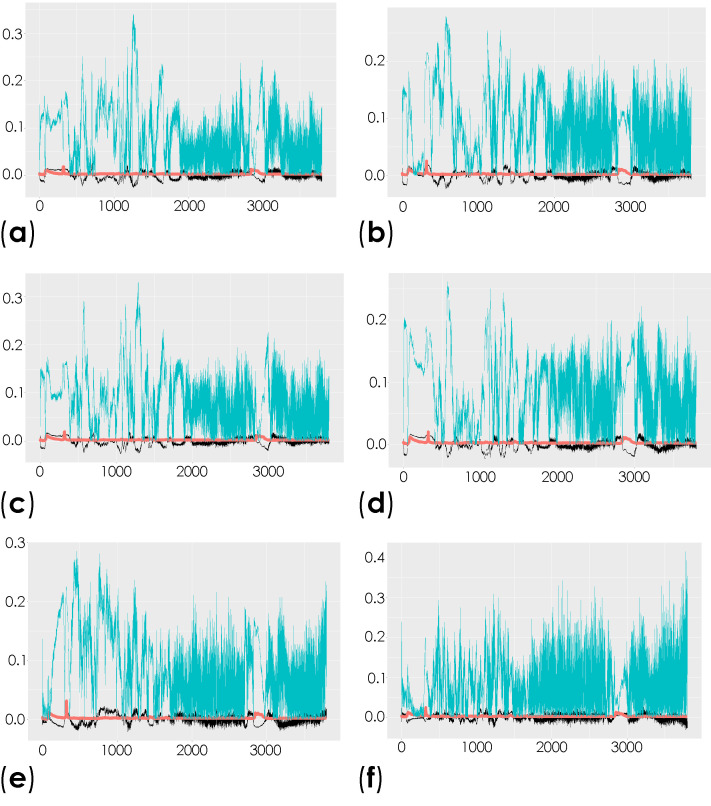

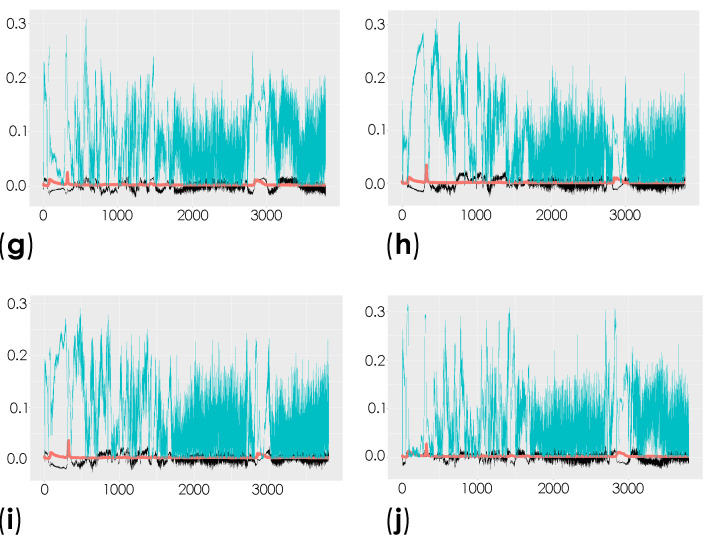
Representation of the coefficient loading plots of the Raman spectra of the unlabeled dECM samples. Different groups were compared: control versus saline contralateral hemisphere (SAL CT) (**a**); control versus saline ipsilateral hemisphere (SAL IP) (**b**); control versus 6-OHDA-lesioned contralateral hemisphere (PD CT) (**c**); control versus 6-OHDA-lesioned ipsilateral hemisphere (PD IP) (**d**); saline contralateral hemisphere (SAL CT) versus saline ipsilateral hemisphere (SAL IP) (**e**); saline contralateral hemisphere (SAL CT) versus 6-OHDA-lesioned contralateral hemisphere (PD CT) (**f**); saline contralateral hemisphere (SAL CT) versus 6-OHDA-lesioned ipsilateral hemisphere (PD IP) (**g**); saline ipsilateral hemisphere (SAL IP) versus 6-OHDA-lesioned contralateral hemisphere (PD CT) (**h**); saline ipsilateral hemisphere (SAL IP) versus 6-OHDA-lesioned ipsilateral hemisphere (PD IP) (**i**); 6-OHDA-lesioned contralateral hemisphere (PD CT) versus 6-OHDA-lesioned ipsilateral hemisphere (PD IP) (**j**).

To investigate the molecular changes in the striatum associated with the induced depletion of the dopaminergic neurons, we compared the total spectra obtained from the decellularized striatum sections (unlabeled samples (spectral range from 0 to 3800 cm^−1^)) of the control mice and the contralateral and ipsilateral hemispheres of saline- and 6-OHDA-lesioned mice using multivariate analysis through OPLS-DA. This analysis is able to reveal the characteristic differences between the two groups [58]. The respective scatter plots of the OPLS-DA scores for all of the comparisons are shown in Figure S2 and Table S1. R2X and R2Y represent the variance explained by the model, while Q2Y indicates the variance predicted by the model (Table S1). In these analyses, we found moderate differences in the comparison of conditions. The corresponding loadings plots showed the peaks responsible for the separation of the groups (Figure 4 and Table S2).

**Figure 5:**
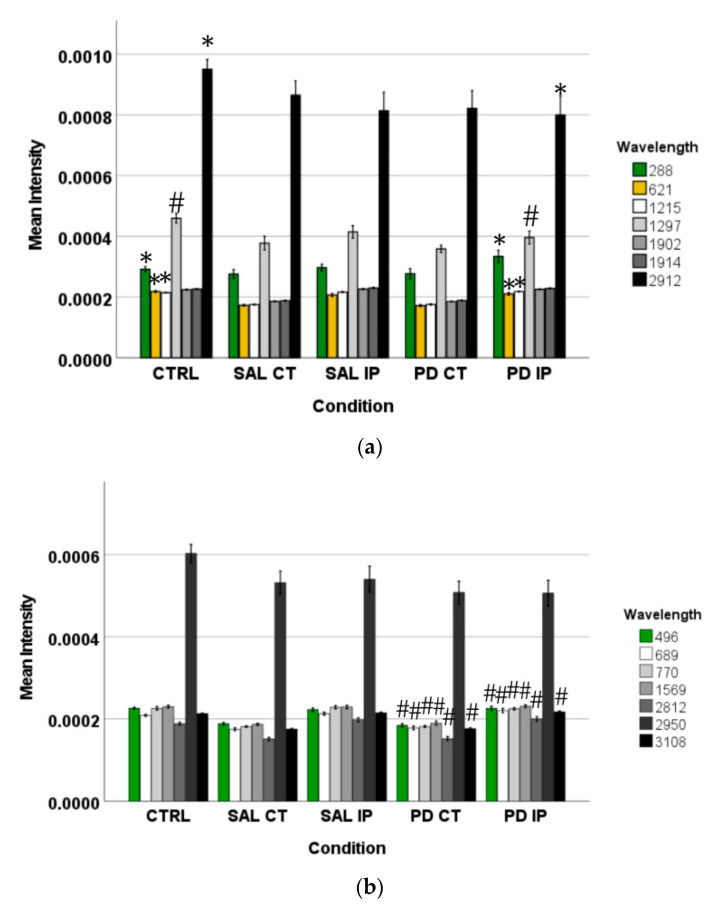

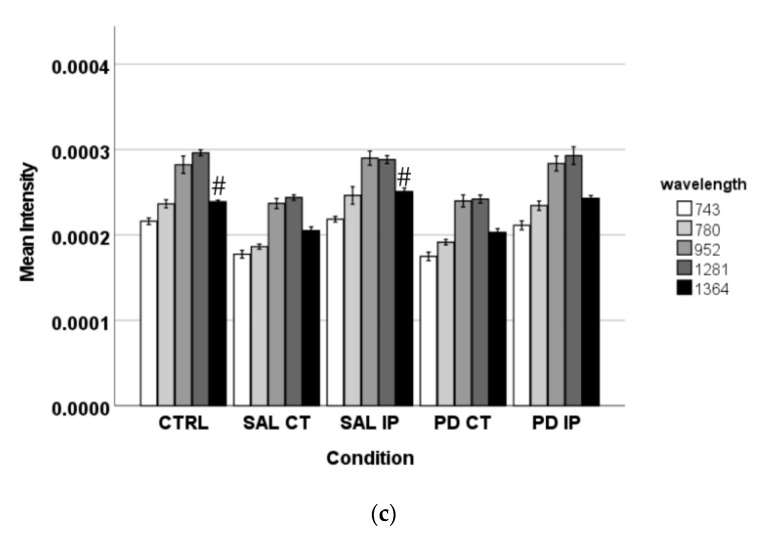
The intensity of the peaks responsible for the separation of the different groups of unlabeled samples found by OPLS-DA was measured in the corresponding Raman spectra. The graphs represent their mean intensity ± SEM from the spectra acquired in the interval from 0 to 3800 cm^−1^. (**a**) Values for the unique peaks appearing in the comparison between control (CTRL) and the ipsilateral hemisphere of the 6-OHDA-lesioned mice (PD IP). (**b**) Values for the unique peaks appearing in the comparison between both hemispheres of the 6-OHDA-lesioned mice (PD CT and PD IP). (**c**) Values for the unique peaks appearing in the comparison between control (CTRL) and the ipsilateral hemisphere of the saline mice (SAL IP). One-way ANOVA or Welch’s ANOVA followed by Bonferroni or Games-Howell *post hoc* test, respectively, was performed, with * *p* < 0.05 and # *p* < 0.001. The sampling distribution was first tested for normality using the Shapiro-Wilk test and for homogeneity of variances using the Levene’s test.

Peaks in the following regions of the spectra resulted from these comparisons: Region I, with the peaks in the interval from 100 to 800 cm^−1^ corresponding to the deformation modes of CCO groups; Region II, with the interval from 800 to 1200 cm^−1^ associated with the stretching vibration of the O-C/C-C groups; Region III, with the interval from 1200 to 1500 cm^−1^ corresponding to the deformation modes of CH/CH_2_ groups; Region IV, with the interval from 1500 to 2400 cm^−1^ correlating with lipids, fatty acids, and the amide I-III bands; and Region V, with the interval from 2700 to 3050 cm^−1^ associated with the stretching vibration of the CH/CH_2_ groups. To show the logical relationships between all of the peaks obtained from the different comparisons, we used a Venn diagram (Table S3). Using this approach, we found seven peaks, 288, 621, 1215, 1297, 1902, 1914, and 2912 cm^−1^, that clearly occurred in the comparisons between the control (CTRL) and the ipsilateral hemisphere of the 6-OHDA-lesioned mice (PD IP; Figure 5). Using one-way ANOVA or Welch’s ANOVA followed by post hoc tests (Bonferroni or Games-Howell, respectively), a comparison was made between the intensities of these peaks for all of our conditions (Table S4). In these comparisons, only the peaks at 288, 621, 1215, 1297, and 2912 cm^−1^ showed significant differences. Assignments in small wavelength regions are rare in the literature, but peaks in the region around 280 cm^−1^ occur in naturally occurring oxalates [59,60]. For our peak at 288 cm^−1^, the condition with the highest intensity was actually the decellularized samples from the ipsilateral hemisphere of the 6-OHDA-lesioned mice (PD IP; Figure 5a). Interestingly, calcium oxalate precipitates are a metabolite from ascorbate and dopamine oxidation produced by hydrogen peroxide, and recently, the increase of oxalate precipitates in the *Substantia nigra* of Parkinson’s disease patients has been described [61]. The other six peaks unique to toxin-treatment have been assigned to the protein peak, phenylalanine [25,48]; amide III [62]; fatty acids, phospholipids, and amide III [25,48,49]; vibration of the C=C bond; and the CH band of lipids and proteins [48].

In addition, the peaks that occurred only from the separation of both hemispheres from the 6-OHDA-lesioned could indicate changes occurring in both hemispheres due to the lesion. These peaks correspond to the wavelengths: 496, 689, 770, 1569, 2812, 2950, and 3108 cm^−1^ (Figure 5b). Statistical analysis was performed using one-way ANOVA or Welch’s ANOVA (followed by a *post hoc* test (Bonferroni or Games-Howell, respectively)) (Table S4). In these comparisons, peaks 496, 689, 770, 1569, 2812, and 3108 cm^−1^ showed significant differences. The assignments to these peaks correspond to: Glycogen; ring deformation; phosphatidylinositol; amide II; CH, CH_2_, and CH_3_ symmetric and asymmetric stretching; CH_3_ asymmetric stretch, CH_3_ stretching vibrations; and CH stretching [48].

Finally, the changes found in the intensity of the peaks at 743, 780, 952, 1281, and 1364 cm^−1^ could be related to the effect of surgical intervention (Figure 5c). To compare these peaks, ANOVA or Welch’s ANOVA (followed by a Bonferroni or Games-Howell post hoc test, respectively) were performed for this comparison (Table S4). In this case, only the peak at 1364 cm^−1^ showed statistical differences for this comparison. This peak was attributed to the CH_3_ symmetrical deformation vibration of lipids [63].

#### 3.2.3. ECM Oxidative Fingerprint and Protein Carbonylation in a Mouse Model of Parkinson’s Disease

The central nervous system is particularly vulnerable to oxidative stress because of its high oxygen consumption. In addition, extracellular components such as collagen, elastin, and proteoglycans accumulate damage during aging in the form of post-translational modifications (PTMs) [64]. It has been shown that Parkinson’s disease patients exhibit increased levels of oxidative stress in their brain, showing lipid and protein oxidation, and comparable events occur in the 6-OHDA mouse model of the disease [3,65]. In the literature, several described Raman peaks have been attributed to modifications of biological macromolecules due to oxidative stress, e.g., carbonyls and advanced glycation end products (AGEs; Table S5) [66,67,68,69,70,71,72,73,74,75,76,77].

Comparisons of the intensities of the oxidation peaks found in the literature revealed differences between groups for all peaks by Welch’s ANOVA or one-way ANOVA, followed by post hoc tests (Games-Howell or Bonferroni test, respectively; Table S6). However, in these comparisons, we did not find a peak that showed a clear effect of the toxin on the oxidative modifications of the ECM. This is because although three peaks (831, 1342, and 1360 cm^−1^) showed differences between the matrices from the ipsilateral hemispheres of the 6-OHDA-lesioned and saline mice, they showed no differences from the control condition. There were also many peaks that showed differences between the ipsilateral hemisphere of the 6-OHDA-lesioned mice and the control condition, but showed no differences between the matrices from the ipsilateral hemispheres of the 6-OHDA-lesioned and the saline mice.

**Figure 6:**
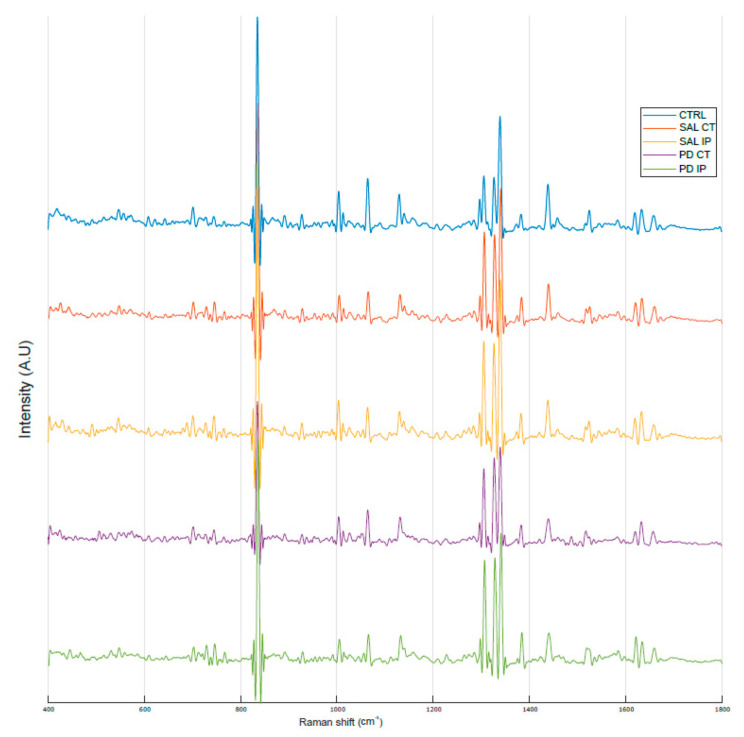
Raman oxidative fingerprint from the average spectra of striatal dECM samples. The spectral interval used for acquisition was from 400 to 1800 cm^−1^. For representation purposes, Matlab software was used to obtain the average smoothed spectra with background subtraction for the striatal decellularized matrices from the control mice (CTRL; total of 57 spectra in blue), saline contralateral hemisphere (SAL CT; total of 48 spectra in orange), saline ipsilateral hemisphere (SAL IP; total of 47 spectra in orange), 6-OHDA-lesioned contralateral hemisphere (PD CT; total of 26 spectra in yellow), and 6-OHDA-lesioned ipsilateral hemisphere (PD IP; total of 42 spectra in green).

Evaluation of protein carbonylation is a recognized marker of oxidation and protein damage [78]. Carbonyl groups can be formed during oxidation of the amino acids of proline, arginine, lysine, and threonine during the oxidative cleavage of proteins, or as a secondary reaction of the nucleophilic side chains of cysteine, histidine, and lysine residues [79]. Since our previous data on the oxidative modifications of the unlabeled matrices were inconclusive, to assess whether the oxidative modifications of the extracellular matrix were present in this animal model of the disease, we incubated the decellularized matrices with 2,4-dinitrophenylhydrazine (DNPH) [31] and analyzed their Raman spectra in the range of 400 to 1800 cm^−1^ (Figure 6).

**Figure 7:**
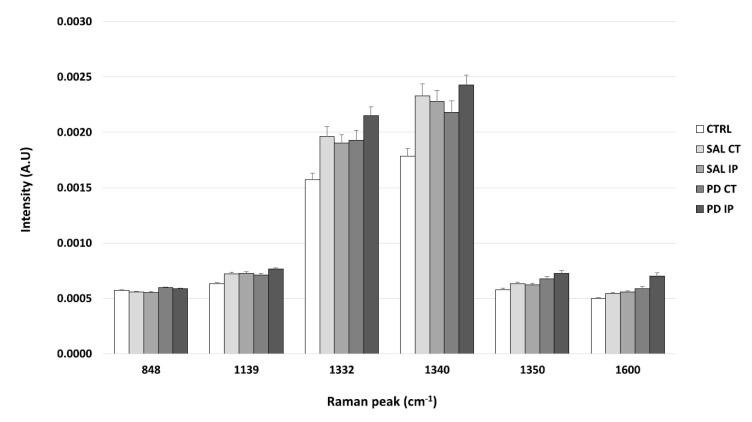
The intensity of the Raman peaks assigned to the DNP in the literature was calculated from the collected Raman spectra from all of the conditions. Mean intensities ± SEM from each condition are presented for the DNP-labeled samples.

It is known that DNPH can conjugate with protein carbonyls, and several authors have found different positions for the DNP specific peaks in a Raman spectrum [31,80,81]. The peaks found in the literature for the DNP are described at 1355 cm^−1^, 1332 cm^−1^, and 1340 cm^−1^ (the peaks attributed to the dinitrophenyl-N bond stretch [31,80,81]); at 848 cm^−1^ (for the ring breathing mode [31]); at 1139 cm^−1^ (for the carbon/DNPH adduct [81]); and at 1600 cm^−1^ (for the C-N-N-C linkage stretch [81]). Using one-way ANOVA or Welch’s ANOVA followed by post hoc tests (Bonferroni or Games-Howell, respectively), a comparison was made between the intensities of these peaks for all of our conditions (Figure 7 and Table S7). Only one peak (at 1350 cm^−1^) showed significant differences uniquely associated with the toxin effect (and not due to the surgical procedure), in both hemispheres of the 6-OHDA-lesioned mice, considering that no differences amongst these hemispheres were found. Another peak (at 1600 cm^−1^) showed differences due to toxin and surgical intervention between the hemispheres of the 6-OHDA-lesioned mice, with the ipsilateral hemisphere (PD IP) displaying the highest value, while for the peaks at 1139, 1332, and 1340 cm^−1^, differences due to surgical intervention were observed, with the ipsilateral hemisphere of the 6-OHDA-lesioned mice showing the highest values.

### 3.3. Cells Growing on the Extracellular Matrix from 6-OHDA-Lesioned Mice Exhibited Decreased Cytoskeletal Tension

The extracellular matrix is a dynamic structure subject to constant remodeling. Cells perceive any changes in its mechanical, structural, and chemical properties, and respond to the changes in the matrix in order to survive and function properly, thus maintaining tissue homeostasis [82]. Through specific matrix receptors located at the plasma membrane, such as integrins, cells detect the changes in the mechanical properties of the extracellular matrix. The activation of these receptors then transmits the information through their association with the intracellular structures of the cytoskeleton, i.e., actin filaments [83]. It is also known that the information of the extracellular matrix stiffness is translated into the reinforcement of the integrin−cytoskeleton axis [84]. In this regard, to address the effect of matrix−cell interactions in the disease context, we used the actinin-sstFRET mechanosensor, which can translate mechanical forces into optical signals [85]. This mechanosensor is based on the protein actinin, which binds to actin and promotes its binding to the plasma membrane.

**Figure 8:**
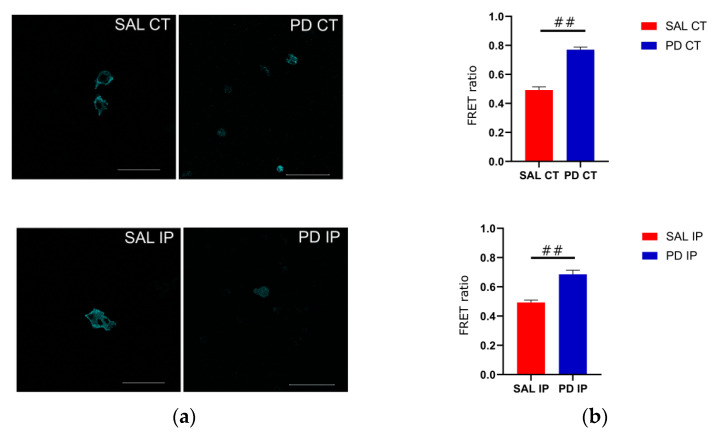
Cells that grew on the striatal decellularized matrices (Scheme 2). (**a**) OS cells expressing the actinin-sstFRET mechanosensor and growing on the decellularized matrices of the contralateral and ipsilateral hemispheres from the saline and 6-OHDA-lesioned mice. The cerulean (donor) image is shown; scale bar 100 μm. (**b**) Comparison of the calculated FRET ratio for all of the conditions, cells growing in the contralateral and ipsilateral hemispheres from the saline (SAL CT and SAL IP) and 6-OHDA-lesioned (PD CT and PD IP) mice. Values are shown indicating the mean value + SEM. One-way ANOVA followed by Bonferroni post hoc test was performed (## *p* < 0.001). The sampling distribution was first tested for normality using the Shapiro−Wilk test and for the homogeneity of variances using the Levene’s test.

Because of its optimal transfection rate, we used the cell line U-2 OS for transfecting the actinin-sstFRET plasmid. After transient transfection, transfected cells were used to repopulate the decellularized matrices from the contralateral and ipsilateral hemispheres of the saline and 6-OHDA-lesioned mice. Images of the donor, FRET, and acceptor were acquired, and the FRET efficiencies were calculated (Figure 8). Actinin-sstFRET-transfected cells that repopulated the matrices from the 6-OHDA-lesioned mice showed significantly higher FRET values when compared to the FRET values of transfected cells repopulated onto the matrices from the saline mice using Welch’s ANOVA, followed by the Games-Howell post hoc test (*p* < 0.00001). This may reflect a lower cytoskeletal fiber load in the cells growing on the matrices of the 6-OHDA-lesioned mice. In addition, there were no significant differences between the FRET values of the transfected cells growing in the contralateral and ipsilateral matrices from the saline mice, suggesting that the mechanical damage to the ipsilateral region was not translated into an altered environment. Regarding the FRET values for the transfected cells that grew in the matrices from the 6-OHDA-lesioned mice, no statistical differences were found between the contralateral and ipsilateral matrices. This suggests that the changes to the extracellular matrix of the ipsilateral and contralateral regions in the 6-OHDA-lesioned mice may negatively affect cell adhesion.

### 3.4. Morphological Evaluation of Primary Microglia in Repopulated Decellularized Matrices

It is now clear that inflammation plays a fundamental role in Parkinson’s disease, as demonstrated not only in animal models of the disease, but also in studies using post-mortem brain samples from patients [86,87]. Activated microglia are recognized key players of Parkinson’s disease, supported by the discovery that the stimulation of these cells with the bacterial lipopolysaccharide (LPS) was sufficient to trigger selective loss of dopaminergic neurons [87]. Microglia cells continuously sense their environment and are capable of being activated in response to detected changes [88]. When these cells are activated, changes occur in their secretome profile and morphology [89]. Interestingly, the shape of microglial correlates with their expression profile, which defines their functional status [90].

**Figure 9:**
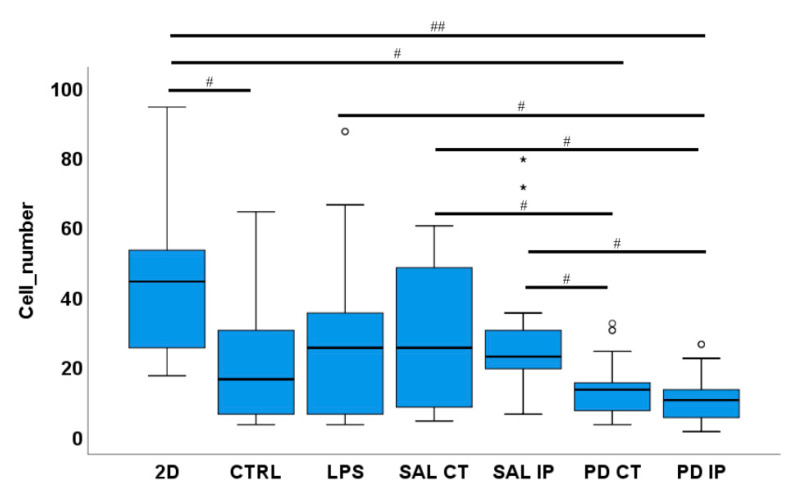
A low number of microglia cells were found in the repopulated matrices from the 6-OHDA-lesioned mice. Microglia cells were seeded onto the striatal decellularized matrices of the control animals (CTRL) and of the contralateral and ipsilateral hemispheres of saline and 6-OHDA-lesioned mice (SAL CT, SAL IP, PD CT, and PD IP, respectively). In addition, microglia cells were seeded onto 2D surfaces (with and without LPS stimulation). The number of microglia cells were counted after 48 h in culture. Values are shown as a box indicating the minimum, maximum, and mean values together with the SEM. Welch’s ANOVA followed by Games Howell post hoc test was performed (# *p* < 0.05 and ## *p* < 0.001). Outliers are marked with the symbols ° and *. The sampling distribution was tested for normality using the Shapiro−Wilk test and for homogeneity of variances using the Levene’s test.

**Figure 10:**
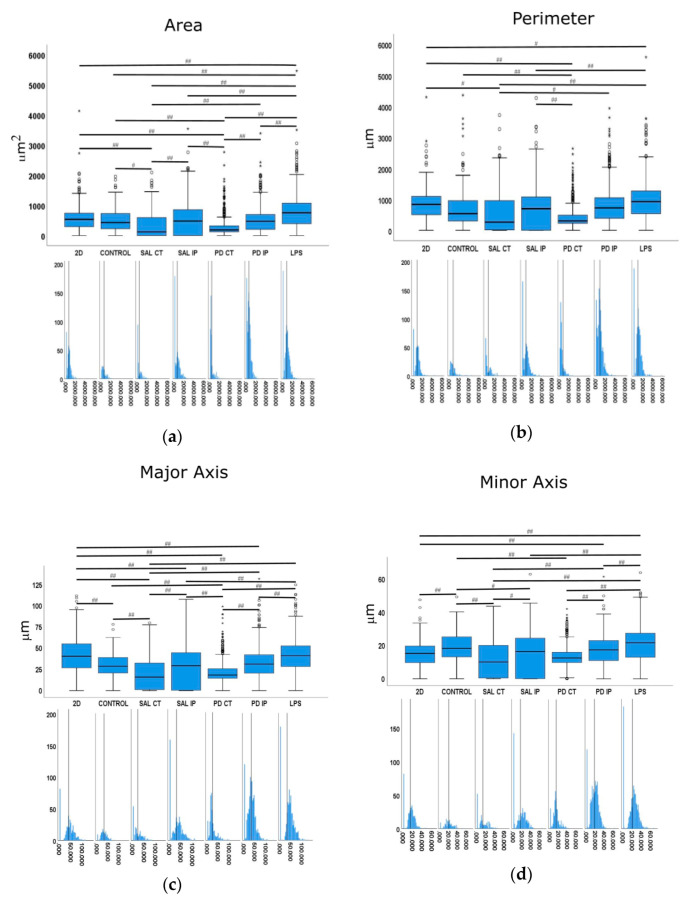

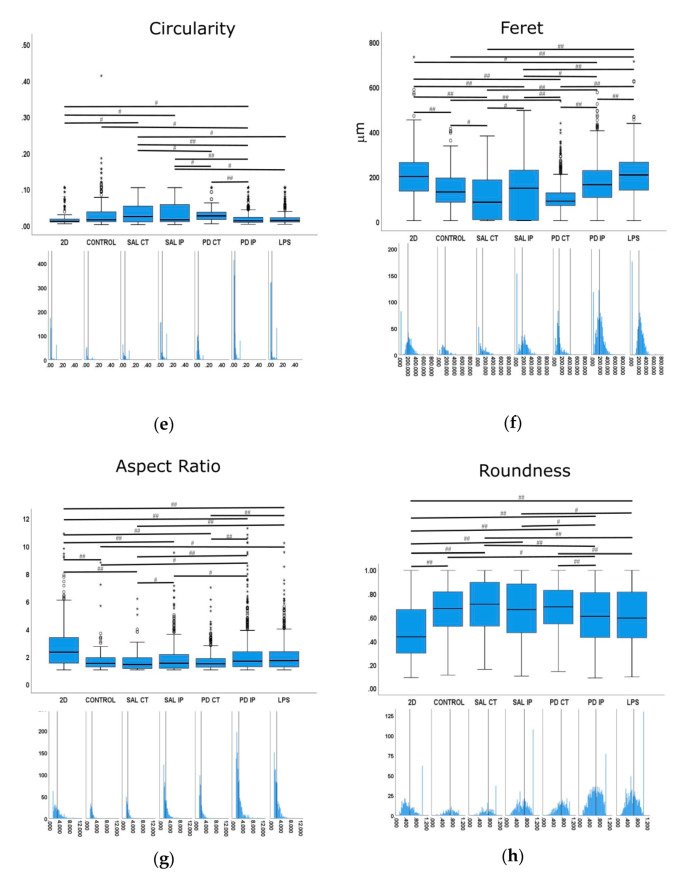

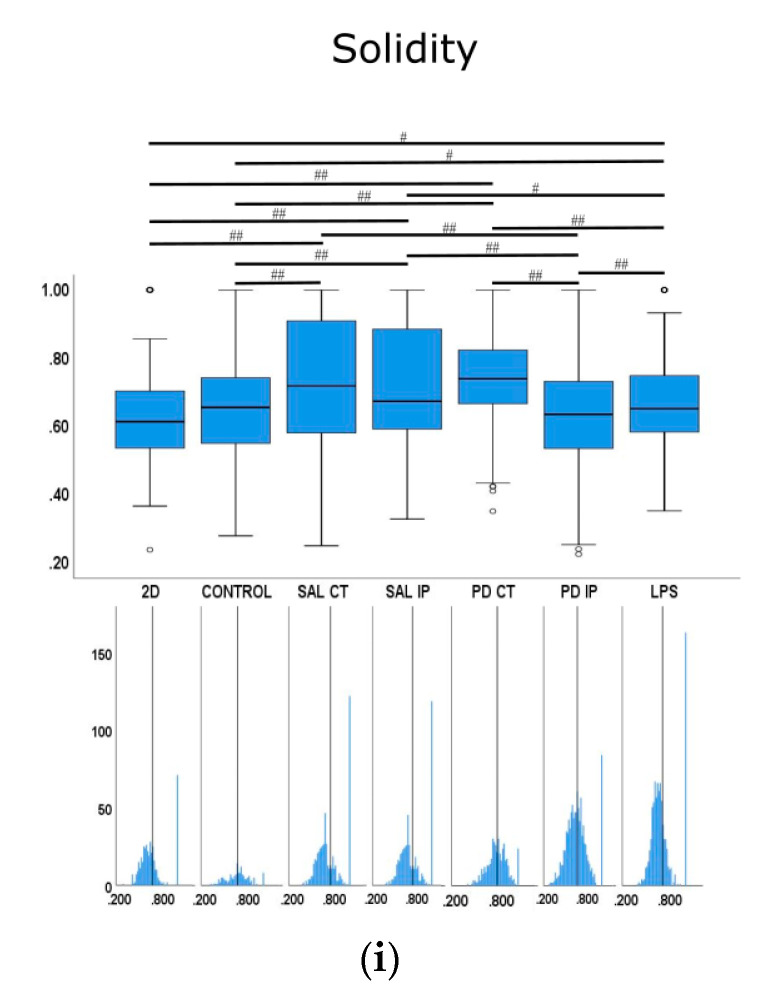
Quantitative morphometric analysis of microglia growing in repopulated striatal decellularized matrices. Nine shape descriptors are quantified for microglia growing in the decellularized matrices from the control animals (CONTROL), matrices from the contralateral and ipsilateral hemispheres of saline and 6-OHDA-lesioned mice (SAL CT, SAL IP, PD CT, and PD IP, respectively), and 2D surface (with and without LPS stimulation). Area (**a**), perimeter (**b**), major axis (**c**), minor axis (**d**), circularity (**e**), Feret’s diameter (**f**), aspect ratio (**g**), roundness (**h**), and solidity (**i**). The values for the corresponding feature are shown as a box displaying the maximum, minimum, and mean ± SEM values. Welch’s ANOVA followed by Games Howell post hoc test were performed (# *p* < 0.05 and ## *p* < 0.001). Outliers are marked with the symbols ° and *. The sampling distribution was tested for normality using the Shapiro−Wilk test and for homogeneity of variances using the Levene’s test. Frequency distribution plots for individual cells are shown below each boxplot, with the vertical line indicating the respective population mean.

To investigate whether the chemical modifications that we found on the extracellular matrix of the 6-OHDA disease model are able to induce morphological changes in microglial cells, we repopulated the decellularized striatal slices with primary cultures of microglia. After two days in culture, we assessed both the ability of the microglia to repopulate the decellularized matrices (Figure 9) and the morphology of microglia by quantifying various shape parameters (Figure 10). Microglia that grew on the decellularized matrices of the control mice and microglia that grew in 2D plastic surfaces (with and without LPS-induced activation) were used as the controls.

The ability of microglial cells to repopulate the decellularized matrices was assessed by counting the number of nuclei of IBA-1 positive microglia cells present in the decellularized matrices 48 h after seeding. For each condition, the mean number of cells ± SEM per field of view was as follows: 46.54 ± 4.78 (2D without LPS), 28.62 ± 4.79 (LPS, cells growing in 2D), 19.42 ± 3.38 (CTRL), 28.77 ± 4.13 (SAL CT), 29.81 ± 4.10 (SAL IP), 13.58 ± 1.54 (PD CT), and 10.35 ± 1.46 (PD IP) (Figure 9). There were statistically significant differences between groups by Welch’s ANOVA (*p* < 0.00001). A Games-Howell post hoc test revealed that in the decellularized matrices from the 6-OHDA-lesioned mice, microglial cells had reduced survival. However, this situation was not statistically significant from the repopulated control matrices.

Furthermore, no statistical differences were observed between the number of primary microglia cells growing in the contralateral and the ipsilateral decellularized matrices of the 6-OHDA-lesioned mice (PD CT versus PD IP; *p*= 0.728), although PD-IP-derived dECM had a lower number of cells. An effect due to the injection of the toxin affecting the ECM of both hemispheres, or the compensatory mechanisms acting on the contralateral side and affecting its ECM, could be possible explanatory mechanisms for this finding [91]. Interestingly, there were differences in the number of microglia cells that grew in the repopulated contralateral and ipsilateral matrices of both the saline and the 6-OHDA-lesioned mice, suggesting that the environment of the matrices of the 6-OHDA-lesioned mice did not favor microglial adhesion or survival, which is consistent with the information obtained from the experiments from the intracellular mechanosensors.

We next examined whether microglia morphology differed in repopulated dECMs from diseased and control mice by quantifying the following nine shape descriptors: area, perimeter, major axis, minor axis, circularity, Feret’s diameter, aspect ratio, roundness, and solidity (Figure 10). Because variances were inhomogeneous between groups (Levene’s test), we compared the group means by Welch’s ANOVA, followed by Games-Howell post hoc test to determine which differences between groups were significant for all of the quantified parameters.

Following an injury event, the activation of the microglia is accompanied by a dramatic change in their morphology, transforming from the branched phenotype of the so-called quiescent microglia to an amoeboid phenotype [89]. In our control for activated microglia (cells growing in 2D surfaces incubated with LPS), we observed that the shape features analyzed showed statistical differences in comparison with the cells growing in 2D without stimulation. These features included area, perimeter, minor axis, roundness, and solidity, which were increased upon LPS stimulation, as well as aspect ratio, which decreased upon stimulation.

In the case of the area descriptor, the maximum mean value was observed in the microglia growing on 2D surfaces with LPS stimulation (LPS), while the smallest value was obtained from microglia cells growing in the matrices of the contralateral side of 6-OHDA-lesioned mice and saline mice (PD CT and SAL CT). Interestingly, microglia cells growing in control dECMs showed no statistically significant differences from the dECMs of the ipsilateral side of both saline (SAL-IP) and 6-OHDA-lesioned (PD-IP) mice, while there were no differences between the microglia growing in saline and 6-OHDA-lesioned mice (SAL IP and PD IP). However, no differences were found between the microglia that grew on the control matrices (CTRL) and those seeded on the ipsilateral region of the 6-OHDA-lesioned mice (PD IP).

For the descriptor perimeter, again the cells stimulated with LPS showed the highest mean value, whereas the microglia growing on the matrices of the contralateral side of the 6-OHDA-lesioned and saline mice showed the smallest value. In addition, there were differences between the contralateral and ipsilateral hemispheres of the 6-OHDA-lesioned and saline mice, respectively. However, no differences were found between the ipsilateral hemispheres. In addition, no differences were found between the cells growing on the control matrices (CTRL) and those that were seeded on the ipsilateral region of the 6-OHDA-lesioned mice (PD IP).

For the minor axis feature, the highest mean value was displayed by the microglia cells that grew in 2D with the LPS stimulus (LPS). No differences were found between the cells that grew on the control matrices (CTRL) and those seeded on the ipsilateral region of the 6-OHDA-lesioned mice (PD IP). Moreover, no differences were found between the ipsilateral hemispheres of the saline and 6-OHDA-lesioned mice (SAL IP and PD IP).

In the case of the roundness feature, the highest mean value was obtained in microglia seeded on the matrices of the contralateral side of the saline mice (SAL CT), while the smallest value was obtained in the cells growing on the 2D surface. Interestingly, statistically significant differences were obtained in the cells that grew in the matrices between the contralateral and ipsilateral sides of the 6-OHDA-lesioned mice (PD CT and PD IP), while there were no differences in the case of saline mice (SAL CT and SAL IP). Although there were differences between the saline and 6-OHDA-lesioned mice (SAL IP and PD IP), no differences were observed between the cells that grew on the contralateral sides (SAL CT and PD CT). In addition, no differences were observed between the cells that grew on the ipsilateral side of the 6-OHDA lesion mice and the LPS-stimulated cells (PD IP and LPS), whereas there were differences between the latter and the cells that grew on the contralateral side of the 6-OHDA-lesioned mice (LPS and PD CT). Overall, the values of this feature support the idea that the microglia cells that grew in the ipsilateral hemisphere of the 6-OHDA mice may perceive an altered matrix.

For the solidity feature, the more branched the object, the larger the convex area, which translates into a smaller solidity value [89]. For this feature, the condition with the highest mean value was the microglia growing on the matrices of the contralateral side of the 6-OHDA-lesioned mice (PD CT), while the condition with the smallest mean value was microglia growing on the ipsilateral side of the 6-OHDA-lesioned mice (PD IP). Interestingly, the mean value of this feature showed statistical significance for the microglia growing on the ipsilateral and contralateral side of the 6-OHDA-lesioned mice (PD CT and PD IP) and between the ipsilateral sides of saline and 6-OHDA-lesioned mice (SAL IP and PD IP). In contrast, no statistical differences were observed between the microglia growing on the contralateral side of saline and 6-OHDA mice (SAL CT and PD CT). Overall, they point in the same direction as the roundness feature.

In the case of the aspect ratio feature, the longer the cell, the higher this value, as shown by the microglia growing on the 2D surface, while the smallest mean value was presented by the cells growing in the matrices on the contralateral side of the saline mice (SAL CT). Both the contralateral and ipsilateral sides of the 6-OHDA-lesioned mice showed a statistically significant difference in their mean values (PD CT and PD IP), and the same occurred with the saline mice (SAL CT and SAL IP). Interestingly, although there were no differences between the contralateral sides of the saline and 6-OHDA-lesioned mice (SAL CT and PD CT), there were differences between the ipsilateral sides (SAL IP and PD IP). In addition, the aspect ratio mean values of the microglia growing on the control matrices showed statistical differences only between the microglia growing on the 2D surfaces and stimulated with LPS and the ipsilateral region of the 6-OHDA-lesioned mice (PD IP). Overall, this suggests that the matrix may undergo changes due to the 6-OHDA lesion conditioning the response of the microglia.

## 4. Discussion

It is widely accepted that oxidative stress plays a significant role in the pathobiology of Parkinson’s disease and that this leads to specific changes in the cellular components of brain cells. However, the changes in the brain extracellular matrix remain poorly understood in the context of this disease, notwithstanding the known relevance of the effects of its surface chemical composition and mechanical properties on cell survival and physiology [92,93]. The present study provides new insight into the modifications of the brain extracellular matrix in a mouse model of Parkinson’s disease. The chemical fingerprint of the decellularized striatum from this animal model was examined by Raman spectroscopy because of the low solubility and high content on the high molecular weight components of the brain’s extracellular matrix. In this regard, our data provide the first biomolecular characterization of the brain’s extracellular matrix in the striatum in an animal model of the disease. This contributes to our understanding of the changes that occur in the diseased brain, as well as expanding our knowledge of the mechanisms that influence the continuous death of the dopaminergic neurons and the relatively low survival of transplanted cells [32].

No previous study has used Raman spectroscopy to analyze the decellularized ECM of the striatal brain in a mouse model of Parkinson’s disease. However, some studies have used this technique for brain tissue analysis. In this regard, Ong and co-workers recorded the Raman spectra of the *Substantia nigra* in a monkey model of Parkinson’s disease [25]. They showed that there is a different peak profile for the white and for the grey matter of the *Substantia nigra* in the range of the spectra from 2800 to 3000 cm^−1^. They showed that in the white matter, the peak profile of this region correlates with a higher lipid content with longer CH_2_ chains (the peaks at 2850 and 2882 cm^−1^ showed a higher intensity than the peak at 2931 cm^−1^, with the peak at 2882 cm^−1^ depicting the highest intensity), while in the grey matter, the most prominent peak in this region was found at 2931 cm^−1^, which corresponds with the CH_3_ groups. However, in the case of our decellularized matrices from the striatum, we found a profile in which the peak at 2850 cm^−1^ was the most prominent (Figure 3). A peak at 2848 cm^−1^ was assigned to the stretching vibrations of CH_2_ and CH_3_ in phospholipids, cholesterol, and creatine (Table 1). Of note, in a comparative study of the Raman fingerprint spectra of different carbohydrates, a peak at 2853 cm^−1^ was found specifically in galactose [46], which is interesting given that the brain ECM is highly enriched in glycosylated macromolecules [94]. Galactose, a monosaccharide sugar, is present in several glycosaminoglycan chains found in chondroitin sulfate, dermatan sulfate, heparin, and heparan sulfate [95], known proteoglycans in the brain [95]. Peaks assigned to carbohydrates (glucose, galactose, and glycogen) in the five carbohydrate domains defined by Wiercigroch et al. were also detected in the Raman spectra of our decellularized matrices. These domains are represented by the stretching vibration of OH (3600–3050 cm^−1^), the stretching vibration of the CH/CH_2_ (3050–2800 cm^−1^), the deformation modes of CH/CH_2_ (1500–1200 cm^−1^), the stretching vibration of the O-C/C-C groups (1200–800 cm^−1^), and the deformation modes of CCO groups (800–100 cm^−1^), some with small shifts (Table 1) [46,48].

An additional indication of the presence of these glycosylated macromolecules is the fact that several other peaks appeared in the spectra from the decellularized matrices, which are common to hyaluronic acid, chondroitin sulfate, and the proteoglycan aggrecan (Table 1) [51]. In the extracellular matrix, the proteoglycan aggrecan, whose side chains are largely composed of chondroitin sulfate, can form an organized network by binding to hyaluronan [96]. Interestingly, aggrecan is a key organizer of the extracellular matrix in the brain, and it is present in perineuronal nets [96]. In addition, in the Raman spectra of the decellularized matrices, a peak appeared that was assigned to the amino acid valine, which is known to be one of the main amino acids of the aggrecan core protein, along with serine and glutamic acid, amino acids that are not present in collagens [51].

Other molecules assigned to the peaks detected in the Raman spectra of our decellularized matrices corresponded to lipids; phospholipids and fatty acids; specific for the lipid cholesterol and cholesterol esters; proteins; and collagen (see Table 1).

Lipids are present in high amounts in lipoproteins, and this type of protein is also present in the extracellular matrix. The most abundant lipoproteins in the brain are the apolipoproteins E and J [97]. The accumulation of lipoproteins in the extracellular matrix may be due to their ability to bind proteoglycans [98]. Comparing the Raman spectra from our decellularized matrices with the characteristic spectra of triglyceride-rich lipoprotein particles [53], we found that most of the Raman signatures for the lipoproteins appeared in our spectra (sometimes with slight shifts) [53] (Table 1). Moreover, in the case of Alzheimer’s disease, it has been suggested that modifications of the lipid structure might occur in the brain of these patients as a result of oxidative stress [57], which is also a common feature of Parkinson’s disease [3].

In addition, Nguyen et al. (2012) compared the Raman spectra of collagen type I and IV. Most of the peaks mentioned in this study appeared in the Raman spectra of our samples (some with slight shifts) [50] (Table 1). Interestingly, the S-S and C-S bond vibrations appear mainly in collagen IV (peaks at 510, 540, and 722 cm^−1^), in contrast with collagen I. Remarkably, collagen IV is the predominant form of collagen found in the brain [99].

Of note, in agreement with our results, some of the above detected peaks were also identified in a mouse model of acute brain injury, albeit with small shifts, namely 1002, 1090, 1130, 1274, 1301, 1450, and 1660 cm^−1^ [55]. These authors found differences between the acutely injured and healthy cortex in the peaks assigned to amide I, with reduced intensities in the injured cortex and the detection of four peaks at 1175, 1227, 1586, and 1618 cm^−1^ in the spectra of the injured cortex. Interestingly, these latter peaks, which could be attributed to cell content as they were associated with cell death, did not appear in our Raman spectra from the decellularized matrices after a decellularization process (Figure 3, Table 1). Other fingerprint assignments for brain tissue found by Huang and co-workers also appeared in our analysis [54] (Table 1).

When comparing the Raman spectra of the unlabeled samples from all of the groups, we found moderate differences between them. This suggests changes in the extracellular matrix caused both by the surgical procedure, probably due to the mechanical injury caused by the injection and the resulting inflammation in the brain, and by the effect of the toxin, which led to the selective degeneration of the dopaminergic neurons. Changes in the Raman spectra that occurred at the brain extracellular matrix in the Parkinson’s disease mouse model included differences in C-H and N-H stretching modes, CH ring, C_1_-C_α_ bending, and CH_2,6_ in-plane bend. In addition, there were also differences in the extracellular matrix components such as polysaccharides; carbohydrates; collagen; amino acids proline, hydroxyproline, tyrosine, and valine; and increased oxalate content. Taken together, these results suggest changes in the components or their structure, arrangements, or interactions of ECM components (i.e., protein aggregation) that could translate into a functional impact. Interestingly, oxalate formation in vivo may occur either through ascorbate and dopamine metabolism, or through dysfunction of the enzyme glyoxylate reductase, which is present in the mitochondria and cytoplasm of all cell types [61]. Both mechanisms may play a role in Parkinson’s disease, as affected cells produce and release the neurotransmitter dopamine and, in addition, mitochondria dysfunction is known to occur [100]. The deficient removal of glyoxylate could lead to its oxidation to oxalate by the enzyme lactate dehydrogenase [101]. Oxidized oxalate may then be deposited in the form of microparticles. Interestingly, the deposition of microparticles of calcium oxalate has been reported in the brain and meninges of patients with primary hyperoxaluria with focal microglial reaction [102], and in the brain of Parkinson’s disease patients [61]. It is known that these crystalline particles of calcium oxalate can activate the inflammasome [61], induce oxidative stress by activating the NADPH enzyme, and cause mitochondria dysfunction, which then contributes to cell damage [103,104]. Interestingly, the enzyme NADPH oxidase, localized at the plasma membrane of microglial cells facing the extracellular space, is responsible for the production of reactive oxygen species, and has been implicated in the pathogenesis of several neurodegenerative diseases, including Parkinson’s disease [105]. Other effects of calcium oxalate crystals on cells include changes in the gene expression, cytoskeletal reorganization, enhancement of matrix composition regulators, and induction of local inflammation [106]. The deleterious effect of these crystals on neurons has also been highlighted by reported axonal loss and demyelination in peripheral nerves [106]. The increased oxalate content that we found in the diseased ECM by Raman spectroscopy therefore needs further investigation to understand whether it contributes directly or indirectly to the oxidative ECM changes found in our study. Nevertheless, the long half-lives of ECM components [107], the lower antioxidant capacity of the extracellular space compared with that of the intracellular space [108], and the permanent damage to macromolecules by oxidative stress [104] might contribute to the accumulation of these oxidative changes. The increased oxidation of the extracellular matrix of the striatum in the 6-OHDA mouse model of the disease was also illustrated by the increase in the specific detection of the post-translational modification carbonylation, a recognized marker of oxidative stress [78]. By using Raman spectroscopy with the DNP-labelled extracellular matrices, we found the highest levels of this adduct in the brain hemisphere where neuronal degeneration occurred. This supports the hypothesis that the matrix of the degenerating hemisphere had higher levels of modifications due to oxidative stress; which is in agreement with the described increased oxidation of the intracellular components of these animal models of the disease [109]. Interestingly, our Raman spectroscopy data also showed that the extracellular matrix of the contralateral hemisphere of the 6-OHDA-lesioned mice also exhibited modifications, suggesting that the disease may have an additional effect on the contralateral hemisphere.

It is now known that the chemical composition, structure, and mechanical properties of the extracellular matrix influence the mechanical properties of the cellular cytoskeleton, the regulation of tissue pattern, and even cell survival [83,110]. Cellular matrix receptors, located at the cell membrane, serve to sense the mechanical stresses exerted on cells [83]. Therefore, the changes in the extracellular matrix identified in the Raman spectra are expected to reflect the surface composition of the matrix and thus alter its mechanical properties. As the sample consistency of the striatum slices was severely compromised after the enrichment protocol, it was not possible for us to directly quantify the mechanical properties of the decellularized striatum using atomic force microscopy or rheometry. Therefore, an indirect measurement of the mechanical properties of the extracellular matrix was used here as an alternative method. We then quantified the mechanical strain on the cytoskeleton of the cells growing on the matrices. By using FRET-based mechanosensors, we showed that the cytoskeleton of the cells growing in the decellularized matrices of the 6-OHDA-lesioned mice exhibited a lower cytoskeletal strain than cells growing in the hemispheres from the saline mice. However, there were no significant differences between the two brain hemispheres of the diseased animal. This is not surprising, as we found that changes in the extracellular matrix due to oxidation occurred in both hemispheres, as shown by our Raman spectroscopy analysis. This may suggest that although the matrix of the ipsilateral hemisphere has different or additional modifications than that of the matrix of the contralateral hemisphere, the modifications occurring in both hemispheres may be sufficient to alter the cellular behavior of the cells growing on these matrices. Of note, our Raman analysis showed an increase in the number of modifications due to oxidative stress and toxic oxalates in the ipsilateral region of the 6-OHDA-lesioned mice. However, no differences were found in the mechanical response of the cells to these modifications between contralateral and ipsilateral regions. There were also no differences in the ability of the primary microglia to repopulate these matrices, suggesting that the modifications may affect cell physiology and survival equally in both hemispheres.

Microglia cells are the resident macrophages of the central nervous system and are considered key players in the disease process. Moreover, their activation by bacterial lipopolysaccharide (LPS) has been shown to model the disease [87]. Activated microglia can release proinflammatory factors that can have deleterious effects on neurons [87]. Moreover, the importance of neuroinflammation in the progression of Parkinson’s disease is well stablished [111]. Microglia cells can exhibit either a quiescent or an activated phenotype, which has been correlated with their morphology [90]. The values of the parameters of area, perimeter, minor axis, aspect ratio, roundness, and solidity showed statistical differences in the microglia growing on the control 2D surfaces compared with the LPS-treated conditions, suggesting that these values are possible descriptors of microglia activation. From the morphometric values of the microglia cells that grew in the ipsilateral region of the decellularized matrices from the 6-OHDA-lesioned mice, it was clear that they presented a bigger perimeter compared with the cells that grew on the other decellularized matrices. They also exhibited a larger minor axis, higher aspect ratio, and lower roundness and solidity, indicating a more branched phenotype than the microglia that grew in the other decellularized matrices, and consequently a more activated phenotype. Moreover, all of these parameters showed significant differences from the cells that grew in the contralateral region of the 6-OHDA-lesioned mice, consistent with the increased soma enlargement and sprouting typical of activated microglial [89]. This may reflect differences in the matrices of the hemispheres that could influence microglial activation along with cell attachment or survival in the lesion area.

## 5. Conclusions

By using confocal Raman microspectroscopy to study the striatal extracellular matrix in a mouse model of Parkinson’s disease, our results indicate post-translational modifications such as carbonylation and the presence of oxalates on the diseased extracellular matrix that directly affect microglia survival and activation, and are also reflected in the increased cytoskeletal stress. We believe that these results help to explain the limited success of cell replacement therapies. Further analysis is needed to identify new avenues for therapies, including neuronal cells therapies in combination with key extracellular matrix components that could support the initial steps of cell survival or the further neuronal connectivity of implanted neurons. A decellularized substrate based on the extracellular matrix as a possible adjuvant for cellular therapies might require the administration of immunosuppressants to counteract the effects of activated microglia, which is already envisioned for stem cell grafts in spinal cord injury models [112].

Our data, besides uncovering possible deleterious modifications of the extracellular matrix in a Parkinson’s disease mouse model, it also points to modifications of the matrix due to the surgical process itself, indicating that the results obtained through this animal model should be carefully evaluated.

## Figures and Tables

**Table 1:** Raman peaks detected in the average spectra from the decellularized matrices (unlabeled samples) acquired in the spectral region from 0 to 3800 cm^−1^. Known peaks from vibrational assignments are included (some with small shifts).

Peaks (cm^−1^)	Assignments	References
322	CaF_2_	[44,45]
404	Found in D(^+^)−glucose and glycogen	[46]
420	C-C=O in-plane deformation. Cholesterol. Found in D(^+^)−glucose and D(^+^)−galactose	[46,47,48]
429	Cholesterol. Cholesterol ester	[48,49]
432	Found in glycogen	[46]
457	Ring torsion of phenyl	[48]
490	Glycogen. Found in D(^+^)−galactose	[46,48,49]
516	S-S vibration of type IV collagen	[50]
519	Phosphatidylinositol. Found in aggrecan	[48,49,51]
546	Cholesterol content. Found in chondroitin sulfate. S-S vibration of type IV collagen	[48,49,50]
556	Beta(OCO), Beta(CCC), Beta(CCO). Found in D(^+^)−glucose	[46]
570	C-C=O in-plane deformation. Cholesterol. Tryptophan/cytosine. Guanine	[47,48]
593	Beta(CCC), ring deformation. Found in glycogen	[46]
608	Cholesterol. Ring deformation of phenyl	[48,49,52]
622	Protein peak. Phenylalanine	[25,48]
625	(600–900 cm^−1^) CH out-of-plane bending vibrations	[48]
643	C-C twisting mode of tyrosine. Found in aggrecan monomers and aggregates	[25,48,49,51,52]
654	630–70 cm^−1^ ν(C–S) gauche (amino acid methionine). Found in D(^+^)−glucose	[46,48]
667	C-S stretching mod of cysteine (collagen type I). Found in D(^+^)−galactose	[46,48,49]
700	ν(C-S) trans (amino acid methionine). C-N stretching mode from the choline head in phosphatidylcholine and sphingomyelin. Found in D(^+^)−galactose	[25,46,48,49,52]
720	C-N (membrane phospholipid head/ nucleotide peak. Symmetric stretch of choline group N^+^(CH_3_)_3_. Characteristic of phospholipids. Phosphatidylcholine. Sphingomyelin. DNA. δ(=C-H) in-plane in triglyceride-rich lipoproteins	[48,49,52,53]
804	Left-handed helix DNA (Z form)	[48]
817	C-C stretching (collagen assignment). More intense in collagen type I	[48,49,50,52]
829	C_2’_ conformation of sugar. Tyrosine	[25,48]
830	Proline. Hydroxyproline. Tyrosine. n_2_ PO_2_ (^−^) stretch of nucleic acids. Found in D(^+^)−galactose	[25,46,48,49,52]
838	Deformative vibrations of amine groups. Found in D(^+^)−glucose	[46,48,49,52]
850	Most probably due to single bond stretching vibrations for the amino acids and valine and polysaccharides. Tyrosine (Fermi resonance of ring fundamental and overtone). Single bond stretching vibrations for the amino acids and valine and polysaccharides. Tyrosine. C-C stretching. Found in aggrecan and chondroitin sulfate. More intense in collagen type IV	[25,48,49,50,51,52]
875	Antisymmetric stretch vibration of choline group N^+^ (CH_3_)_3_. Characteristic for phospholipids. Phosphatidylcholine. Sphingomyelin. ν(C-C) hydroxyproline (protein assignment). C-C stretching hydroxyproline (collagen assignment). More intense in collagen type IV	[25,48,49,50,52]
891	Saccharide band (overlaps with acyl band). Found in D(^+^)−galactose. Found in hyaluronic acid and chondroitin sulfate	[46,48,49,51,52]
928–40	ν(C-C) stretching probably in amino acids proline and valine (protein band). More intense in collagen type IV	[48,49,50]
938	Proline. Hydroxyproline. n(C-C) skeletal of collagen backbone. C-C stretch backbone. Found in glycogen.	[46,48,49,52]
950–3	Most probably due to single-bond stretching vibrations for the amino acids proline and valine and polysaccharides. CH_6′.5′_ out of plane	[48]
961	Tryptophan ring breathing; C–O deoxyribose. C–C. Found in D(^+^)−galactose. Appears in brain tissue	[46,49,54]
972	C-C backbone (collagen assignment). OCH_3_ (polysaccharides. pectin). Found in D(^+^)-galactose. DNA. δ(=C-H) out of plane in triglyceride-rich lipoproteins	[46,48,49,52,53]
989	Ring stretch modes of benzene derivatives. Appears in brain tissue. Phenylalanine NADH	[48,54]
996	C-O ribose. C-C. Carbohydrates peak for solutions and solids. Carbohydrates (weak shoulder). Found in D(^+^)−galactose	[46,48,49]
1004	Phenylalanine (of collagen; protein assignment). νs(C-C). Symmetric ring breathing. Phenyl breathing mode. ν(C-C) phenylalanine. Found in D(^+^)−glucose. More intense in collagen type IV. Decreased at the injured brain	[46,48,49,50,52,55]
1012	CHα.α’ out-of-plane bending and Cα= Cα’ torsion	[48]
1064	Skeletal C-C stretch of lipids. Acyl chains. ν(C-C) trans. stretching C-O ribose. CO stretching and C-O-C stretching and PO2 symmetric stretch. Found in D(^+^)−galactose. Found in chondroitin sulfate, and aggrecan monomers and aggregates. ν(C-C) in triglyceride-rich lipoprotein particles	[25,46,48,49,51,52,53]
1074	Glucose. Triglycerides. C-C (lipid). Found in D(^+^)-glucose. ν(C-C) in triglyceride-rich lipoprotein particles	[46,49,53]
1085	ν(C-C) gauche. PO_2_ asymmetric stretching of phospholipids and nucleic acid	[25,52]
1086	ν(C-C) gauche. Symmetric phosphate stretching modes or ν(PO_2_^−^)sym (phosphate stretching modes originate from the phosphodiester groups in nucleic acids and suggest an increase in the nucleic acids in the malignant tissues). PO_2_^−^ symmetric. ν(PO_2_^−^) symmetric stretching of phosphodiesters. Found in glycogen	[46,48,49,52]
1095	Lipid. ν(C-N). Phosphodioxy group (PO_2_^-^ in nucleic acids). Stretching PO_2_(^−^) symmetric. DNA: O–P–O backbone stretching	[48,52]
1130	C-C skeletal stretch trans conformation in lipids. Phospholipid structural changes (trans versus gauche isomerism). Acyl chains. CH_3_ deformations (both symmetric and asymmetric). Found in glycogen	[46,48,49,52]
1268	(=C-H) (phospholipids). Ring stretch. Amide III (collagen assignment). Found in glycogen. δ(=C-H) in plane cis	[46,48,49,52,53]
1274	Amide III. Found in D(^+^)−glucose. More intense in collagen type IV	[46,48,50]
1298	Palmitic acid. Acyl chains. Fatty acids. Phospholipid CH_2_ twist and wagging. amide III (a fibrous). CH_3_/CH_2_ twisting or bending of lipid/collagen	[25,48,49,54]
1438	Lipid. δ(CH_2_) δas(CH_3_) in lipid. CH_2_ deformation. δ(CH_2_) scissor in triglyceride-rich lipoprotein particles. Fatty acids, CH_2_ (lipids and proteins)	[25,48,49,53,54]
1452	CH_2_ bending mode of proteins. CH_2_CH_3_ deformation. CH_2_ deformation in lipids. δ(CH_2_) in triglyceride-rich lipoprotein particles	[48,49,53]
1458	CH_3_ deformation. CH_2_ deformation. Nucleic acid modes. DeltaCH_3_ of collagen. Amide (III) stretch. Found in D(^+^)−glucose and glycogen	[46,48,49,52,56]
1549	Amide II of proteins.	[48,54]
1657	Fatty acids. Amide I (collagen assignment). Triglycerides (fatty acids). α-helical structure of amide I. ν(C=C) cis (lipid), and ν(O-H) (water) in triglyceride-rich lipoprotein particles. Decreased in the injury brain	[25,48,49,52,53,55]
1670	Amide I. C=C stretching vibrations. Cholesterol & its esters C=C stretching vibration mode of steroid ring; Amide I (anti-parallel β-sheet). ν(C=C) trans. Lipids. Fatty acids.	[27,48,49,52]
2330	Atmospheric N_2_	[49,54]
2341	Asymmetric stretching band of CO_2_− hydrates	[48]
2677	Stretching N-H(NH_3_^+^)	[48]
2704	2700–3000 C-H stretches. Stretching vibrations of CH. NH. and OH groups	[48]
2711	2700–3000 C-H stretches. Stretching vibrations of CH. NH. and OH groups	[48]
2719	2700–3000 C-H stretches. Stretching vibrations of CH. NH. and OH groups	[48]
2727	Stretching N-H (NH_3_^+^)	[48]
2734	2700–3000 C-H stretches. Stretching vibrations of CH. NH. and OH groups	[48]
2768	Stretching N-H(NH_3_+)	[48]
2775	2700–3000 C-H stretches. Stretching vibrations of CH. NH. and OH groups	[48]
2848	Cholesterol. Phospholipids and creatine (higher in normal tissues). Stretching vibrations of CH_2_ and CH_3_ of phospholipids. Cholesterol and creatine. Found in D(^+^)−galactose. CH_2_ symmetric stretching vibrations	[25,46,48,57]
2861	Stretching C-H	[48]
2865	C=O	[48]
2868	Symmetric stretching of methoxy (3)	[48]
2874	νCH_3_. stretching C-H. N-H. CH_3_ symmetric stretch of lipids; CH_2_ symmetric stretch of lipids	[48,49,54]
2882	Stretching C-H. Asymmetric vibration of CH_2_. Found in D(^+^)−glucose	[25,46,48]
2891	Stretching C-H. Found in D(^+^)−glucose	[46,48]
2898	CH_3_ symmetric stretch	[48]
2904	CH_3_ stretching modes (both symmetric and asymmetric)	[48]
2910	CH_3_ stretching vibrations. Found in glycogen	[46,48,49]
2915	CH band of lipids and proteins. Found in D(^+^)−galactose. Appears in brain tissue	[46,48,49,54]
2939	Asymmetric stretching of methoxy(2). Symmetric vibration of CH_3_. Found in D(^+^)−glucose. CH_3_ symmetric stretching vibrations	[25,46,48,57]
2952	CH_3_ stretching vibrations. CH_3_ asymmetric stretch	[48]
2959	C-H stretching. νCH_3_ of lipids. DNA and proteins. Asymmetric stretching mode of the methyl groups from cellular proteins. Nucleic acids and lipids. Asymmetric stretching of methoxy (2)	[25,48]
2960	νasCH_3_; Out-of-plane chain-end antisymmetric CH_3_ stretch band	[48,49]
2964	CH_3_ modes. Found in D(^+^)−glucose	[46,48]
2971	Asymmetric stretching of methoxy(4)	[48,49,52]
2975	Stretching N-H. Stretching C-H. Found in D(^+^)−galactose	[46,48,49]
2982	C-H	[48]
2990	C-H ring/ C-H	[48]
3005	3000–600 cm^−1^ C-H stretching	[48]
3012	Unsaturated = CH stretch	[48]
3018	CH_2′_ aromatic stretch	[48]

## Data Availability

The data presented in this study are available on request from the corresponding author.

## Supplementary Materials

The following are available online at https://www.mdpi.com/article/10.3390/antiox10071095/s1. Figure S1: Tyrosine hydroxylase (TH) protein expression in mouse striatal brain slices. Figure S2: Multivariate analysis of the spectral region from 0 to 3800 cm^−1^ from the Raman spectra obtained from the unlabeled dECMs. Table S1: OPLS-DA results obtained for the different comparisons between conditions of the collected Raman spectra for the interval from 0 to 3800 cm^−1^ from the unlabeled samples. Table S2: Raman peaks appearing in the coefficient loading plots from the spectra acquired in interval 0–3800 cm^−1^ from the unlabeled samples, Table S3: List of peaks from the coefficient loading plots appearing in each comparison. Table S4: Statistical analysis of the different comparisons between samples of the Raman peaks found in the coefficient loadings plot from the spectra acquired in interval 0–3800 cm^−1^ from the unlabeled samples. Table S5: List of Raman peaks typically assigned as carbonyls and advanced glycation end products (AGEs), and their respective assignments. Table S6: Statistical analysis of the different comparisons from the Raman peaks typically assigned in the literature as carbonyls and advanced glycation end products found in our unlabeled samples. Table S7: Statistical analysis of the DNP Raman peaks described in the literature.
