# Supplementary material for: Characterization of the Striatal Extracellular Matrix in a Mouse Model of Parkinson’s Disease
Source: Antioxidants (Basel). 2021 Jul 8;10(7):1095. doi: 10.3390/antiox10071095 (PMC8301085; doi:10.3390/antiox10071095)
Supplement: Supplementary file 1 [file antioxidants-10-01095-s001.zip › SupplementaryFigures_MLa.pdf]

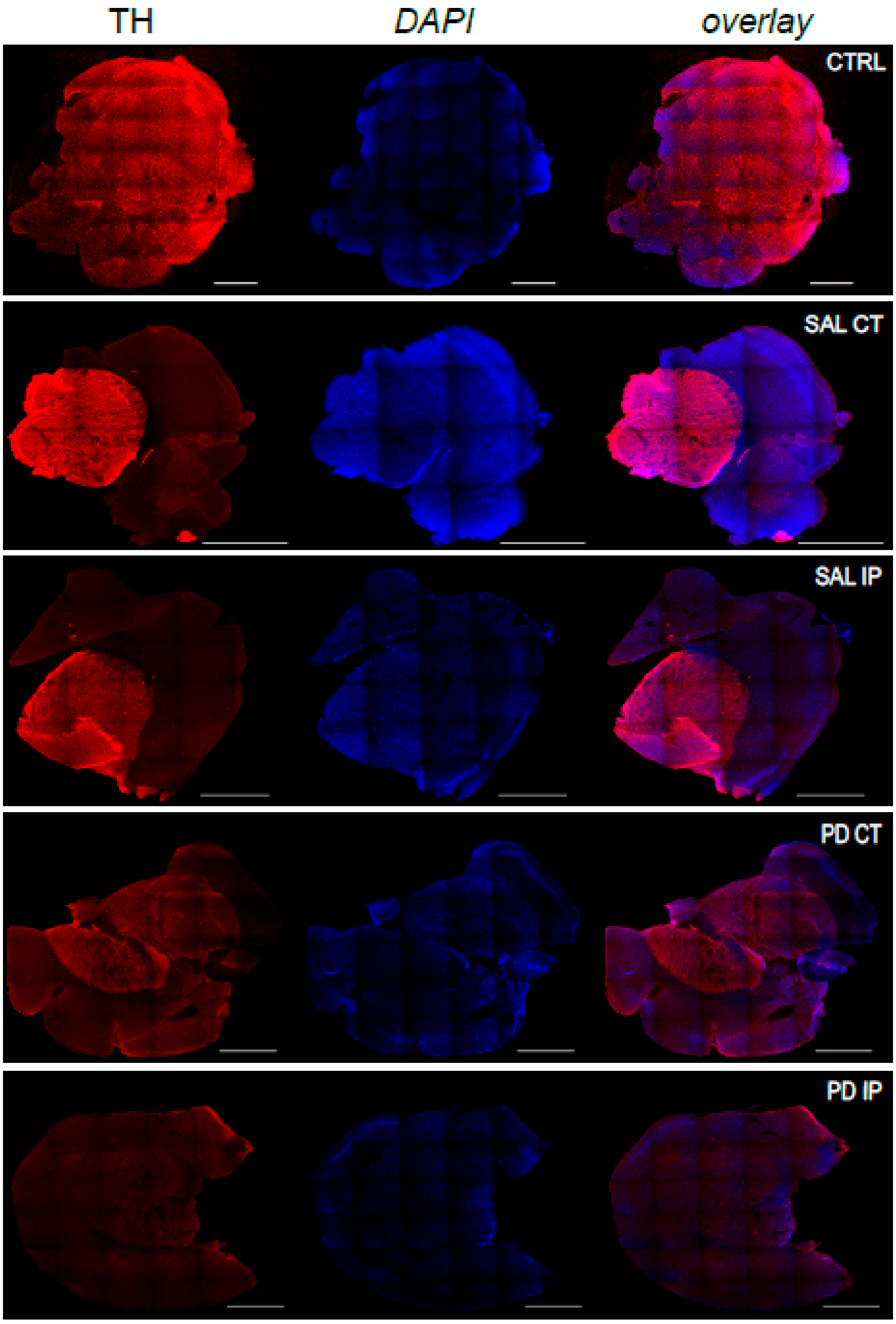

Figure S1. *Tyrosine hydroxylase (TH) protein expression in mouse striatal brain slices. Anti-TH antibody was used to detect the expression of the TH protein (in red) in brain striatal slices extracted from the 6-OHDA-lesioned mice (PD CT and PD IP from the contralateral and ipsilateral hemispheres respectively), and the saline-injected mice (SAL CT and SAL IP from the contralateral and ipsilateral hemispheres respectively), and from control animals (CTRL). As expected from this animal model of the disease, there was a marked reduction of the TH protein expression in the ipsilateral hemisphere of the 6-OHDA-lesioned animals. Nuclei were stained with DAPI (blue). Scale bar 1.7 mm.*

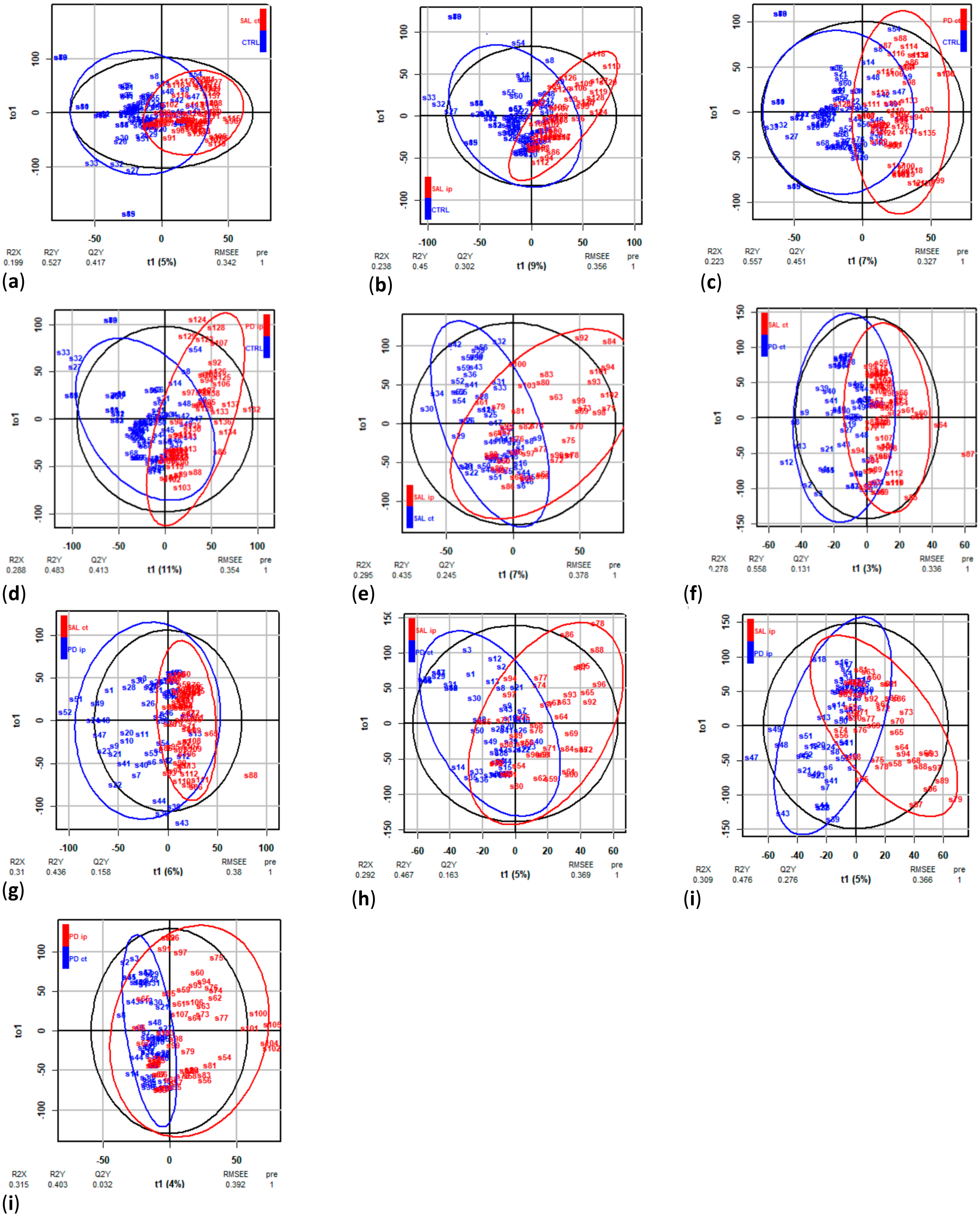

Figure S2. *Multivariate analysis of the spectral region from 0 to 3800 cm^{-1} from the Raman spectra obtained from the unlabeled dECMs. Representation of the OPLS-DA scores plots for the comparisons of the spectra from the decellularized striatal matrices: control versus saline contralateral hemisphere (SAL CT) (**a**); control versus saline ipsilateral hemisphere (SAL IP) (**b**); control versus 6-OHDA-lesioned contralateral hemisphere (PD CT) (**c**); control versus 6-OHDA-lesioned ipsilateral hemisphere (PD IP) (**d**); saline contralateral hemisphere (SAL CT) versus saline ipsilateral hemisphere (SAL IP) (**e**); saline contralateral hemisphere (SAL CT) versus 6-OHDA-lesioned contralateral hemisphere (PD CT) (**f**); saline contralateral hemisphere (SAL CT) versus 6-OHDA-lesioned ipsilateral hemisphere (PD IP) (**g**); saline ipsilateral hemisphere (SAL IP) versus 6-OHDA-lesioned contralateral hemisphere (PD CT) (**h**); saline ipsilateral hemisphere (SAL IP) versus 6-OHDA-lesioned ipsilateral hemisphere (PD IP) (**i**); 6-OHDA-lesioned contralateral hemisphere (PD CT) versus 6-OHDA-lesioned ipsilateral hemisphere (PD IP) (**j**).*
