# Supplementary material for: Characterization of the Striatal Extracellular Matrix in a Mouse Model of Parkinson’s Disease
Source: Antioxidants (Basel). 2021 Jul 8;10(7):1095. doi: 10.3390/antiox10071095 (PMC8301085; doi:10.3390/antiox10071095)
Supplement: Supplementary file 1 [file antioxidants-10-01095-s001.zip › SupplementaryTables_MLa.pdf]

Table S1. OPLS-DA results obtained for the different comparisons between conditions of the collected Raman spectra for the interval 0 to 3800 cm^{-1} from the unlabeled samples. All comparisons showed moderate variance explained by the model, according to the classification: 1 ≥ R2Y ≤ 0.67; 0.67 > R2Y ≤ 0.33; 0.33 > R2Y ≤ 0.19.

	R2X(cum)	R2Y(cum)	Q2(cum)	t1	RMSEE	pre	ort	pR2Y	pQ2
CTRL vs SAL CT	0.199	0.527	0.417	5%	0.342	1	1	0.05	0.05
CTRL vs SAL IP	0.238	0.450	0.302	9%	0.356	1	1	0.05	0.05
CTRL vs PD CT	0.223	0.557	0.451	7%	0.327	1	1	0.05	0.05
CTRL vs PD IP	0.288	0.483	0.413	11%	0.354	1	1	0.05	0.05
SAL CT vs SAL IP	0.295	0.435	0.245	7%	0.378	1	1	0.1	0.05
SAL CT vs PD CT	0.278	0.558	0.131	3%	0.336	1	1	0.05	0.05
SAL CT vs PD IP	0.310	0.436	0.158	6%	0.380	1	1	0.05	0.05
SAL IP vs PD CT	0.292	0.467	0.163	5%	0.369	1	1	0.15	0.05
SAL IP vs PD IP	0.309	0.476	0.276	5%	0.366	1	1	0.3	0.05
PD CT vs PD IP	0.315	0.403	0.032	4%	0.392	1	1	0.55	0.05

Table S2. Raman peaks appearing in the coefficient loading plots from the spectra acquired in interval 0-3800 cm^{-1} from the unlabeled samples.

**Loadings (cm^{-1})**	**CTRL vs SAL CT**	**CTRL vs SAL IP**	**CTRL vs PD CT**	**CTRL vs PD IP**	**SAL CT vs SAL IP**	**SAL CT vs PD CT**	**SAL CT *vs* PD IP**	**SAL IP vs PD CT**	**SAL IP vs PD IP**	**PD CT vs PD IP**
100-800	466<br>476<br>572<br>618<br>651<br>703<br>711<br>779	318<br>412<br>443<br>466<br>476<br>572<br>592<br>743<br>780	412<br>435<br>476<br>524<br>572<br>618<br>644<br>703<br>779	288<br>423<br>466<br>484<br>524<br>572<br>592<br>610<br>621<br>703<br>765	278<br>318<br>412<br>435<br>466<br>500<br>510<br>550<br>572<br>610<br>637<br>644<br>729<br>765	318<br>423<br>476<br>484<br>510<br>550<br>572<br>610<br>618<br>629<br>648<br>651<br>703<br>717<br>774<br>779	318<br>423<br>484<br>550<br>572<br>610<br>651<br>703<br>717<br>779	278<br>318<br>384<br>435<br>466<br>630<br>644<br>651<br>729<br>765	223<br>278<br>318<br>400<br>412<br>443<br>476<br>508<br>550<br>629<br>651<br>765	423<br>466<br>496<br>572<br>689<br>703<br>765<br>770
800 - 1200	823<br>850<br>867<br>877<br>890<br>969<br>995<br>1054<br>1081<br>1123<br>1191	839<br>901<br>952<br>1004<br>1012<br>1123<br>1144<br>1191<br>1199	831<br>850<br>1054<br>1101<br>1123<br>1131<br>1191	823<br>850<br>890<br>995<br>1027<br>1054<br>1081<br>1101<br>1131<br>1191<br>1199	823<br>850<br>867<br>901<br>932<br>1004<br>1012<br>1016<br>1101<br>1144	802<br>823<br>831<br>839<br>901<br>942<br>995<br>1027<br>1043<br>1054<br>1081<br>1114<br>1124<br>1144<br>1172	909<br>991<br>995<br>1054<br>1064<br>1101<br>1123<br>1131<br>1191<br>1199	802<br>850<br>867<br>991<br>995<br>1012<br>1043<br>1101<br>1160	835<br>850<br>877<br>1012<br>1027<br>1054<br>1081<br>1101<br>1110<br>1126<br>1160<br>1191	901<br>1043<br>1054<br>1081<br>1123<br>1191
1200 - 1500	1257	1281	1289	1215	1207	1207	1220	1237	1220	1243

	1275<br>1298<br>1306<br>1309<br>1377<br>1419<br>1438<br>1478<br>1488<br>1489	1289<br>1364<br>1377<br>1382<br>1419<br>1466	1412<br>1449<br>1489	1268<br>1289<br>1297<br>1405<br>1412<br>1466	1220<br>1233<br>1237<br>1243<br>1377<br>1382<br>1428<br>1438<br>1475	1220<br>1237<br>1289<br>1309<br>1314<br>1360<br>1377<br>1412<br>1428<br>1449<br>1475<br>1489	1237<br>1306<br>1309<br>1314<br>1377<br>1419<br>1428<br>1466<br>1478	1257<br>1268<br>1282<br>1331<br>1342<br>1363<br>1370<br>1382<br>1385<br>1428	1268<br>1314<br>1342<br>1363<br>1375<br>1382<br>1428<br>1429<br>1475<br>1489	1289<br>1306<br>1309<br>1405<br>1419<br>1488
1500 - 2400	1593<br>1640<br>1661	1543<br>1797	1640<br>1738<br>2338	1661<br>1902<br>1914	1585<br>1691<br>1694	1543<br>1670<br>1738<br>1771<br>1797	1543<br>1670<br>1771<br>1797	1543<br>1681<br>1738<br>1771<br>1797	1543<br>1661<br>1670<br>1681<br>1738<br>1771<br>1797	1569
2700 - 3110	2827<br>2830<br>2874<br>2927<br>2939<br>3001<br>3050	2749<br>2756<br>2874<br>2966<br>3001<br>3050	2749<br>2833<br>2874<br>2927<br>3001<br>3026<br>3050<br>3054	2756<br>2912<br>2966<br>2977<br>3050	2749<br>2764<br>2799<br>2827<br>2874<br>2966<br>3001<br>3026<br>3029<br>3050	2749<br>2756<br>2833<br>2874<br>2977<br>3001<br>3050	2727<br>2827<br>2966<br>2977<br>3050	2749<br>2756<br>2833<br>2874<br>3001<br>3050	2827<br>2737<br>2874<br>3001<br>3026<br>3050	2749<br>2812<br>2827<br>2874<br>2950<br>2966<br>3050<br>3108

Table S3. *List of peaks from the coefficient loading plots appearing in each comparison. The logical relations were obtained by the open-source Venn diagram software from the Van de Peer Lab, available at http://bioinformatics.psb.ugent.be/cgi-bin/liste/Venn/calculate_venn.htpl*

**Comparisons**	**total**	**Elements**
CTRLvsPDCT CTRLvsPDIP CTRLvsSALCT CTRLvsSALIP PDCTvsPDIP SALCTvsPDCT SALCTvsPDIP SALCTvsSALIP SALIPvsPDCT SALIPvsPDIP	1	3050
CTRLvsPDCT CTRLvsPDIP CTRLvsSALCT CTRLvsSALIP PDCTvsPDIP SALCTvsPDCT SALCTvsPDIP SALCTvsSALIP	1	572
CTRLvsPDCT CTRLvsSALCT CTRLvsSALIP PDCTvsPDIP SALCTvsPDCT SALCTvsSALIP SALIPvsPDCT SALIPvsPDIP	1	2874
CTRLvsPDCT CTRLvsPDIP CTRLvsSALCT CTRLvsSALIP PDCTvsPDIP SALCTvsPDIP SALIPvsPDIP	1	1191
CTRLvsPDCT CTRLvsSALCT CTRLvsSALIP SALCTvsPDCT SALCTvsSALIP SALIPvsPDCT SALIPvsPDIP	1	3001
CTRLvsPDCT CTRLvsPDIP CTRLvsSALCT PDCTvsPDIP SALCTvsPDCT SALCTvsPDIP SALIPvsPDIP	1	1054
CTRLvsPDIP CTRLvsSALCT CTRLvsSALIP PDCTvsPDIP SALCTvsSALIP SALIPvsPDCT	1	466
CTRLvsPDCT CTRLvsPDIP CTRLvsSALCT SALCTvsSALIP SALIPvsPDCT SALIPvsPDIP	1	850
CTRLvsPDCT CTRLvsPDIP CTRLvsSALCT PDCTvsPDIP SALCTvsPDCT SALCTvsPDIP	1	703
CTRLvsPDCT CTRLvsSALIP PDCTvsPDIP SALCTvsPDCT SALCTvsSALIP SALIPvsPDCT	1	2749
CTRLvsSALIP SALCTvsPDCT SALCTvsPDIP SALCTvsSALIP SALIPvsPDCT SALIPvsPDIP	1	318
CTRLvsPDCT CTRLvsPDIP SALCTvsPDIP SALCTvsSALIP SALIPvsPDCT SALIPvsPDIP	1	1101
CTRLvsPDCT CTRLvsSALCT CTRLvsSALIP SALCTvsPDCT SALIPvsPDIP	1	476
CTRLvsPDCT CTRLvsSALCT CTRLvsSALIP PDCTvsPDIP SALCTvsPDIP	1	1123
CTRLvsSALCT CTRLvsSALIP SALCTvsPDCT SALCTvsPDIP SALCTvsSALIP	1	1377

CTRLvsPDIP CTRLvsSALCT SALCTvsPDCT SALCTvsPDIP SALIPvsPDCT	1	995	
CTRLvsPDIP CTRLvsSALCT PDCTvsPDIP SALCTvsPDCT SALIPvsPDIP	1	1081	
CTRLvsSALCT PDCTvsPDIP SALCTvsPDIP SALCTvsSALIP SALIPvsPDIP	1	2827	
CTRLvsSALCT SALCTvsPDCT SALCTvsPDIP SALIPvsPDCT SALIPvsPDIP	1	651	
CTRLvsPDCT CTRLvsPDIP CTRLvsSALIP PDCTvsPDIP SALCTvsPDCT	1	1289	
CTRLvsPDIP CTRLvsSALIP PDCTvsPDIP SALCTvsPDIP SALCTvsSALIP	1	2966	
CTRLvsSALIP SALCTvsPDCT SALCTvsPDIP SALIPvsPDCT SALIPvsPDIP	2	1543	1797
CTRLvsPDIP PDCTvsPDIP SALCTvsSALIP SALIPvsPDCT SALIPvsPDIP	1	765	
SALCTvsPDCT SALCTvsPDIP SALCTvsSALIP SALIPvsPDCT SALIPvsPDIP	1	1428	
CTRLvsSALCT CTRLvsSALIP PDCTvsPDIP SALCTvsPDIP	1	1419	
CTRLvsPDCT CTRLvsSALCT SALCTvsPDCT SALCTvsPDIP	1	779	
CTRLvsPDCT CTRLvsSALCT SALCTvsPDCT SALIPvsPDIP	1	1489	
CTRLvsPDIP CTRLvsSALCT SALCTvsPDCT SALCTvsSALIP	1	823	
CTRLvsSALCT PDCTvsPDIP SALCTvsPDCT SALCTvsPDIP	1	1309	
CTRLvsPDCT CTRLvsSALIP SALCTvsSALIP SALIPvsPDIP	1	412	
CTRLvsPDIP CTRLvsSALIP SALCTvsPDCT SALIPvsPDCT	1	2756	
CTRLvsSALIP PDCTvsPDIP SALCTvsPDCT SALCTvsSALIP	1	901	
CTRLvsSALIP SALCTvsSALIP SALIPvsPDCT SALIPvsPDIP	2	1012	1382
CTRLvsPDCT SALCTvsPDCT SALIPvsPDCT SALIPvsPDIP	1	1738	
CTRLvsPDIP SALCTvsPDCT SALCTvsPDIP SALCTvsSALIP	1	610	
CTRLvsPDIP PDCTvsPDIP SALCTvsPDCT SALCTvsPDIP	1	423	
SALCTvsPDCT SALCTvsPDIP SALCTvsSALIP SALIPvsPDCT	1	1237	
SALCTvsPDCT SALCTvsPDIP SALCTvsSALIP SALIPvsPDIP	2	550	1220
SALCTvsPDCT SALCTvsPDIP SALIPvsPDCT SALIPvsPDIP	1	1771	
CTRLvsPDCT CTRLvsSALCT SALCTvsPDCT	1	618	

CTRLvsPDIP CTRLvsSALCT SALIPvsPDIP	1	1661	
CTRLvsSALCT SALCTvsSALIP SALIPvsPDCT	1	867	
CTRLvsSALCT PDCTvsPDIP SALCTvsPDIP	1	1306	
CTRLvsPDIP CTRLvsSALIP SALCTvsPDIP	2	1199	1466
CTRLvsSALIP SALCTvsPDCT SALCTvsSALIP	1	1144	
CTRLvsPDCT CTRLvsPDIP SALCTvsPDCT	1	1412	
CTRLvsPDCT CTRLvsPDIP SALCTvsPDIP	1	1131	
CTRLvsPDCT SALCTvsSALIP SALIPvsPDCT	2	435	644
CTRLvsPDCT SALCTvsSALIP SALIPvsPDIP	1	3026	
CTRLvsPDCT SALCTvsPDCT SALIPvsPDCT	1	2833	
CTRLvsPDIP SALCTvsPDCT SALCTvsPDIP	2	484	2977
CTRLvsPDIP SALCTvsPDCT SALIPvsPDIP	1	1027	
CTRLvsPDIP SALIPvsPDCT SALIPvsPDIP	1	1268	
SALCTvsPDCT SALCTvsSALIP SALIPvsPDIP	1	1475	
SALCTvsSALIP SALIPvsPDCT SALIPvsPDIP	1	278	
SALCTvsPDCT SALCTvsPDIP SALIPvsPDIP	2	1314	1670
PDCTvsPDIP SALCTvsPDCT SALIPvsPDCT	1	1043	
CTRLvsPDCT CTRLvsSALCT	2	1640	2927
CTRLvsPDIP CTRLvsSALCT	1	890	
CTRLvsSALCT SALCTvsSALIP	1	1438	
CTRLvsSALCT SALCTvsPDIP	1	1478	
CTRLvsSALCT SALIPvsPDCT	1	1257	
CTRLvsSALCT SALIPvsPDIP	1	877	
CTRLvsSALCT PDCTvsPDIP	1	1488	
CTRLvsPDIP CTRLvsSALIP	1	592	
CTRLvsSALIP SALCTvsSALIP	1	1004	
CTRLvsSALIP SALCTvsPDCT	1	839	
CTRLvsSALIP SALIPvsPDIP	1	443	
CTRLvsPDCT CTRLvsPDIP	1	524	
CTRLvsPDCT SALCTvsPDCT	2	831	1449
CTRLvsPDIP PDCTvsPDIP	1	1405	
SALCTvsPDCT SALCTvsSALIP	2	510	1207
SALCTvsSALIP SALIPvsPDCT	1	729	
PDCTvsPDIP SALCTvsSALIP	1	1243	
SALCTvsPDCT SALCTvsPDIP	1	717	

SALCTvsPDCT SALIPvsPDCT	1	802										
SALCTvsPDCT SALIPvsPDIP	1	629										
SALCTvsPDIP SALIPvsPDCT	1	991										
SALIPvsPDCT SALIPvsPDIP	4	1160	1342	1363	1681							
CTRLvsSALCT	7	711	969	1275	1298	1593	2830	2939				
CTRLvsSALIP	5	743	780	952	1281	1364						
CTRLvsPDCT	2	2338	3054									
CTRLvsPDIP	7	288	621	1215	1297	1902	1914	2912				
SALCTvsSALIP	11	500	637	932	1016	1233	1585	1691	1694	2764	2799	3029
SALCTvsPDCT	7	648	774	942	1114	1124	1172	1360				
SALCTvsPDIP	3	909	2727	1064								
SALIPvsPDCT	6	384	630	1282	1331	1370	1385					
SALIPvsPDIP	9	223	400	508	835	1110	1126	1375	1429	2737		
PDCTvsPDIP	7	496	689	770	1569	2812	2950	3108				

Table S4. *Statistical analysis of the different comparisons between samples of the Raman peaks found in the coefficient loadings plot from the spectra acquired in interval 0-3800 cm^{-1} from the unlabeled samples. Either one-way ANOVA or Welch's ANOVA was performed, followed by post hoc tests Bonferroni or Games-Howell respectively. Sample distribution was initially tested for normality using the Shapiro-Wilk test, and Levene's for testing the homogeneity of variances. In bold are highlighted the comparisons for which p<0.05.*

**Wavelength (cm^{-1})**	**Type**	**F**	**p**	***Post Hoc* test**	**p**
288	Welch's ANOVA	10.175	0.0000	Games-Howell	
				CTRL - SAL CT	0.325
				CTRL - SAL IP	0.945
				CTRL - PD CT	0.531
				CTRL - PD IP	**0.003**
				SAL CT - SAL IP	0.133
				SAL CT - PD CT	1.000
				SAL CT - PD IP	**0.000**
				SAL IP - PD CT	0.272
				SAL IP - PD IP	**0.018**
				PD CT - PD IP	**0.000**
496	Welch's ANOVA	132.273	0.0000	Games-Howell	
				CTRL - SAL CT	**0.000**
				CTRL - SAL IP	0.630
				CTRL - PD CT	**0.000**
				CTRL - PD IP	1.000
				SAL CT - SAL IP	**0.000**
				SAL CT - PD CT	0.525
				SAL CT - PD IP	**0.000**
				SAL IP - PD CT	**0.000**
				SAL IP - PD IP	0.824
				PD CT - PD IP	**0.000**
621	One-way ANOVA	142.039	0.0000	Bonferroni	
				CTRL - SAL CT	**0.000**
				CTRL - SAL IP	**0.000**
				CTRL - PD CT	**0.000**
				CTRL - PD IP	**0.020**
				SAL CT - SAL IP	**0.000**
				SAL CT - PD CT	1.000
				SAL CT - PD IP	**0.000**
				SAL IP - PD CT	**0.000**
				SAL IP - PD IP	1.000
				PD CT - PD IP	**0.000**
689	Welch's ANOVA	111.464	0.0000	Games-Howell	
				CTRL - SAL CT	**0.000**

				CTRL - SAL IP	0.418
				CTRL - PD CT	**0.000**
				CTRL - PD IP	**0.001**
				SAL CT - SAL IP	**0.000**
				SAL CT - PD CT	0.840
				SAL CT - PD IP	**0.000**
				SAL IP - PD CT	**0.000**
				SAL IP - PD IP	0.121
				PD CT - PD IP	**0.000**
743	One-way ANOVA	144.998	0.0000	Bonferroni	
				CTRL - SAL CT	**0.000**
				CTRL - SAL IP	1.000
				CTRL - PD CT	**0.000**
				CTRL - PD IP	1.000
				SAL CT - SAL IP	**0.000**
				SAL CT - PD CT	1.000
				SAL CT - PD IP	**0.000**
				SAL IP - PD CT	**0.000**
				SAL IP - PD IP	0.748
				PD CT - PD IP	**0.000**
770	Welch's ANOVA	310.904	0.0000	Games-Howell	
				CTRL - SAL CT	**0.000**
				CTRL - SAL IP	0.915
				CTRL - PD CT	**0.000**
				CTRL - PD IP	0.988
				SAL CT - SAL IP	**0.000**
				SAL CT - PD CT	1.000
				SAL CT - PD IP	**0.000**
				SAL IP - PD CT	**0.000**
				SAL IP - PD IP	0.549
				PD CT - PD IP	**0.000**
780	Welch's ANOVA	148.229	0.0000	Games-Howell	
				CTRL - SAL CT	**0.000**
				CTRL - SAL IP	0.272
				CTRL - PD CT	**0.000**
				CTRL - PD IP	0.989
				SAL CT - SAL IP	**0.000**
				SAL CT - PD CT	0.416
				SAL CT - PD IP	**0.000**
				SAL IP - PD CT	**0.000**
				SAL IP - PD IP	0.474
				PD CT - PD IP	**0.000**
952	One-way ANOVA	37.439	0.0000	Bonferroni	

				CTRL - SAL CT	**0.000**
				CTRL - SAL IP	1.000
				CTRL - PD CT	**0.000**
				CTRL - PD IP	1.000
				SAL CT - SAL IP	**0.000**
				SAL CT - PD CT	1.000
				SAL CT - PD IP	**0.000**
				SAL IP - PD CT	**0.000**
				SAL IP - PD IP	1.000
				PD CT - PD IP	**0.000**
1215	One-way ANOVA	791.787	0.0000	Bonferroni	
				CTRL - SAL CT	**0.000**
				CTRL - SAL IP	0.940
				CTRL - PD CT	**0.000**
				CTRL - PD IP	**0.007**
				SAL CT - SAL IP	**0.000**
				SAL CT - PD CT	1.000
				SAL CT - PD IP	**0.000**
				SAL IP - PD CT	**0.000**
				SAL IP - PD IP	1.000
				PD CT - PD IP	**0.000**
1281	Welch's ANOVA	131.781	0.0000	Games-Howell	
				CTRL - SAL CT	**0.000**
				CTRL - SAL IP	0.053
				CTRL - PD CT	**0.000**
				CTRL - PD IP	0.972
				SAL CT - SAL IP	**0.000**
				SAL CT - PD CT	0.968
				SAL CT - PD IP	**0.000**
				SAL IP - PD CT	**0.000**
				SAL IP - PD IP	0.925
				PD CT - PD IP	**0.000**
1297	One-way ANOVA	20.188	0.0000	Bonferroni	
				CTRL - SAL CT	**0.000**
				CTRL - SAL IP	**0.009**
				CTRL - PD CT	**0.000**
				CTRL - PD IP	**0.000**
				SAL CT - SAL IP	0.102
				SAL CT - PD CT	1.000
				SAL CT - PD IP	1.000
				SAL IP - PD CT	**0.002**
				SAL IP - PD IP	1.000
				PD CT - PD IP	0.078

1364	Welch’s ANOVA	124.880	0.0000	Games-Howell	
				CTRL - SAL CT	**0.000**
				CTRL - SAL IP	**0.000**
				CTRL - PD CT	**0.000**
				CTRL - PD IP	0.187
				SAL CT - SAL IP	**0.000**
				SAL CT - PD CT	0.966
				SAL CT - PD IP	**0.000**
				SAL IP - PD CT	**0.000**
				SAL IP - PD IP	**0.028**
				PD CT - PD IP	**0.000**
1569	One-way ANOVA	146.668	0.0000	Bonferroni	
				CTRL - SAL CT	**0.000**
				CTRL - SAL IP	1.000
				CTRL - PD CT	**0.000**
				CTRL - PD IP	1.000
				SAL CT - SAL IP	**0.000**
				SAL CT - PD CT	1.000
				SAL CT - PD IP	**0.000**
				SAL IP - PD CT	**0.000**
				SAL IP - PD IP	1.000
				PD CT - PD IP	**0.000**
1902	One-way ANOVA	1002.451	0.0000	Bonferroni	
				CTRL - SAL CT	**0.000**
				CTRL - SAL IP	1.000
				CTRL - PD CT	**0.000**
				CTRL - PD IP	1.000
				SAL CT - SAL IP	**0.000**
				SAL CT - PD CT	1.000
				SAL CT - PD IP	**0.000**
				SAL IP - PD CT	**0.000**
				SAL IP - PD IP	1.000
				PD CT - PD IP	**0.000**
1914	One-way ANOVA	653.238	0.0000		
				CTRL - SAL CT	**0.000**
				CTRL - SAL IP	0.059
				CTRL - PD CT	**0.000**
				CTRL - PD IP	0.489
				SAL CT - SAL IP	**0.000**
				SAL CT - PD CT	1.000
				SAL CT - PD IP	**0.000**
				SAL IP - PD CT	**0.000**
				SAL IP - PD IP	1.000

				PD CT - PD IP	**0.000**
2812	Welch's ANOVA	132.592	0.0000	Games-Howell	
				CTRL - SAL CT	**0.000**
				CTRL - SAL IP	**0.027**
				CTRL - PD CT	**0.000**
				CTRL - PD IP	**0.008**
				SAL CT - SAL IP	**0.000**
				SAL CT - PD CT	0.999
				SAL CT - PD IP	**0.000**
				SAL IP - PD CT	**0.000**
				SAL IP - PD IP	0.975
				PD CT - PD IP	**0.000**
2912	Welch's ANOVA	128.906	0.0000	Games-Howell	
				CTRL - SAL CT	**0.027**
				CTRL - SAL IP	**0.002**
				CTRL - PD CT	**0.002**
				CTRL - PD IP	**0.001**
				SAL CT - SAL IP	0.664
				SAL CT - PD CT	0.768
				SAL CT - PD IP	0.463
				SAL IP - PD CT	1.000
				SAL IP - PD IP	0.998
				PD CT - PD IP	**0.986**
2950	One-way ANOVA	10.116	0.0000	Bonferroni	
				CTRL - SAL CT	**0.001**
				CTRL - SAL IP	**0.015**
				CTRL - PD CT	**0.000**
				CTRL - PD IP	**0.000**
				SAL CT - SAL IP	1.000
				SAL CT - PD CT	1.000
				SAL CT - PD IP	1.000
				SAL IP - PD CT	1.000
				SAL IP - PD IP	1.000
				PD CT - PD IP	1.000
3108	One-way ANOVA	560.852	0.0000	Bonferroni	
				CTRL - SAL CT	**0.000**
				CTRL - SAL IP	0.101
				CTRL - PD CT	**0.000**
				CTRL - PD IP	**0.003**
				SAL CT - SAL IP	**0.000**
				SAL CT - PD CT	1.000
				SAL CT - PD IP	**0.000**
				SAL IP - PD CT	**0.000**

				SAL IP - PD IP	1.000
				PD CT - PD IP	**0.000**

Table S5. *List of Raman peaks typically assigned as carbonyls and advanced glycation end products (AGEs), and their respective assignments.*

**Peaks (cm^{-1})**	**Assignment**	**References**
408	Increased upon Abeta peptide (1-40) oxidation	[66]
500-535	S-S stretching vibrations mode on the structure C-C-S-S-C-C	[67]
508-512	S-S stretching band for the TGT conformer	[68]
523-528	S-S stretching band for the GGT conformer	[68]
540-545	S-S stretching band for the GGG conformer	[68]
609	Methyl glyoxal	[69]
671	2-Amino-5-(2-amino-5-hydro-5-methyl-4-imidazolon-1-yl)-pentanoic acid (MG-H2)	[69]
685	Methyl glyoxal	[69]
697	Methionine sulfoxide	[69]
702	vC-S. increased upon Met oxidation	[70]
704	vC-S. increased upon Met oxidation	[71]
705	CS stretching. increased upon Met oxidation	[72]
717	increased upon Met protein oxidation	[72]
724	S=O stretch. Increased upon Abeta peptide (1-40) oxidation	[66]
750	C-S vibration of the Cys residue (decrease upon oxidation)	[67]
801	Pentosidine	[69]
803	Pentosidine	[73]
809	Methyl glyoxal	[69]
831	Methylglyoxal-derived hydroimidazolone-1 (MG-H1)	[69]
836	Acrolein/pentosidine	[69]
838	Pentosidine	[73]
845	Acrolein	[69]
855	Proline	[74]
877	Hydroxyproline	[74]
880	AGE	[73]
890	4-hydroxyhexenal (HHE)	[69]
900	2-Amino adipic acid	[69]
922	Proline	[74]
930	Carboxymethyl lysine	[69]
945	vC-S=O. increased upon Met oxidation	[70]

970	Pentosidine	[73]
980	AGE	[73]
995	Pentosidine	[73]
1003	C-S stretching mode. Increased upon Abeta peptide (1-40) oxidation	[63]
1006	SO stretching. appears upon Met oxidation	[72]
1010	vS=O. Increased upon Met oxidation	[70, 71]
1016	Methionine sulfoxide	[69]
1026	Methionine sulfoxide	[69]
1044	vS=O. Increased upon Met oxidation	[70]
1045	vS=O of oxidized Cys	[70]
1080-1090	AGE	[73]
1134	vS=O. Increased upon Met oxidation	[70]
1150	Carboxymethyl lysine. Pentosidine	[73, 74]
1157	Tyr	[67]
1160	Pentosidine	[73]
1215	Pentosidine	[73]
1300	Pentosidine	[73]
1306	Bending vibration of C-H. Moderate increase upon moderate oxidation, but high decreased in highly oxidized proteins	[67]
1320	Carboxyethyl Lysine/carboxymethyl Lysine	[69]
1326	Pentosidine	[73]
1342	Methylglyoxal-lysine dimer (MOLD)/Glyoxal-lysine dimer (GOLD)	[69]
1360	Carboxymethyl Lysine. Pentosidine	[69, 73]
1362	Pentosidine	[74]
1390	Carboxyethyl Lysine	[69]
1400	Methylglyoxal-lysine dimer (MOLD)	[69]
1405	Carboxyethyl Lysine	[69]
1417	Carboxymethyl lysine/2-Amino adipic acid	[69]
1419	Increased upon Met oxidation	[71]
1420	Glyoxal-lysine dimer (GOLD)	[69]
1428	δCH2. increased upon Met oxidation	[70]
1430	Pentosidine	[73]
1439	Methylglyoxal-lysine dimer (MOLD)	[69]

1445	Glyoxal-lysine dimer (GOLD)	[69]
1450	Bending vibration of CH2 and CH3. Moderate increase upon moderate oxidation, but high decreased in highly oxidized proteins	[67]
1454	Carboxyethyl Lysine/methyl glyoxal	[69]
1455	Pentosidine	[73]
1460	Carboxymethyl Lysine/methylglyoxal-derived hydroimidazolone-1 (MG-H1)	[69]
1496	Pentosidine	[74]
1520	Vibration of the Trp indole ring. Intensity decreases upon oxidation	[67]
1534	4-hydoxyhexenal (HHE)	[69]
1580	Carboxylethyl lysine	[69]
1612	Methylglyoxal-lysine dimer (MOLD)	[69]
1620	Acrolein	[69]
1630	Increased (parallel) Beta-sheet structure and/or of oxidative forms of amino acids side chains	[75]
1640	Stretching vibration of C=O. Increases upon oxidation	[67]
1620-1632	Beta-sheet. Decreases in both moderate and high oxidant conditions	[76]
1635-1639	Beta-turn. Increases both in moderate and high oxidant conditions	[76]
1637	2-Amino-5-(2-amino-5-hydro-5-methyl-4-imidazolon-1-yl)-pentanoic acid (MG-H2)	[69]
1640-1645	Random coil. Increases in moderate oxidant conditions and decreases in high oxidant conditions	[76]
1655	Acrolein	[69]
1650-1665	Beta-sheet. Decreases in moderate and high oxidant conditions	[76]
1666-1670	Random coil. Increases in moderate oxidant conditions and decreases in high oxidant conditions	[76]
1665-1680	Beta-sheet. Decreases in moderate and high oxidant conditions	[76]
1680-1690	Beta-turn. Increases in moderate and high oxidant conditions	[76]
1690	4-hydroxyhexenal (HHE)	[69]
1697	Methylglyoxal-derived hydroimidazolone-1 (MG-H1)/ 2-Amino-5-(2-amino-5-hydro-5-methyl-4-imidazolon-1-yl)-pentanoic acid (MG-H2)	[69]
1734	Methyl glyoxal	[69]
2255	Nitrile group stretch	[77]

Table S6. *Statistical analysis of the different comparisons from the Raman peaks typically assigned in the literature as carbonyls and advanced glycation end products found in our unlabeled samples. Either one-way ANOVA or Welch's ANOVA was performed, followed by post hoc test Bonferroni or Games-Howell, respectively. Sample distribution was initially tested for normality using the Shapiro-Wilk test and Levene's for testing the homogeneity of variances.*

**Wavelength (cm^{-1})**	**Type**	**F**	***p***	***Post Hoc* test**	**p**
408	ANOVA	30.167	0	Bonferroni	
				CTRL - SAL CT	**0.000**
				CTRL - SAL IP	1.000
				CTRL - PD CT	**0.000**
				CTRL - PD IP	**0.010**
				SAL CT-SAL IP	**0.000**
				SAL CT- PD CT	1.000
				SAL CT- PD IP	**0.000**
				SAL IP - PD CT	**0.000**
				SAL IP - PD IP	1.000
				PD CT - PD IP	**0.000**
500	Welch's ANOVA	138.310	0	Games-Howell	
				CTRL - SAL CT	**0.000**
				CTRL - SAL IP	**0.000**
				CTRL - PD CT	**0.000**
				CTRL - PD IP	**0.020**
				SAL CT-SAL IP	**0.000**
				SAL CT- PD CT	1.000
				SAL CT- PD IP	**0.000**
				SAL IP - PD CT	**0.000**
				SAL IP - PD IP	0.960
				PD CT - PD IP	**0.000**
535	Welch's ANOVA	106.444	0	Games-Howell	
				CTRL - SAL CT	**0.000**
				CTRL - SAL IP	**0.010**
				CTRL - PD CT	**0.000**
				CTRL - PD IP	**0.000**
				SAL CT-SAL IP	**0.000**
				SAL CT- PD CT	1.000
				SAL CT- PD IP	**0.000**
				SAL IP - PD CT	**0.000**
				SAL IP - PD IP	0.560
				PD CT - PD IP	**0.000**
609	Welch's ANOVA	53.483	0	Games-Howell	
				CTRL - SAL CT	**0.000**
				CTRL - SAL IP	0.860
				CTRL - PD CT	**0.000**
				CTRL - PD IP	0.580

				SAL CT-SAL IP	**0.000**
				SAL CT- PD CT	0.920
				SAL CT- PD IP	**0.000**
				SAL IP - PD CT	**0.000**
				SAL IP - PD IP	1.000
				PD CT - PD IP	**0.000**
671	Welch's ANOVA	71.434	0	Games-Howell	
				CTRL - SAL CT	**0.000**
				CTRL - SAL IP	0.170
				CTRL - PD CT	**0.000**
				CTRL - PD IP	**0.030**
				SAL CT-SAL IP	**0.000**
				SAL CT- PD CT	0.960
				SAL CT- PD IP	**0.000**
				SAL IP - PD CT	**0.000**
				SAL IP - PD IP	0.980
				PD CT - PD IP	**0.000**
685	Welch's ANOVA	112.180	0	Games-Howell	
				CTRL - SAL CT	**0.000**
				CTRL - SAL IP	**0.000**
				CTRL - PD CT	**0.000**
				CTRL - PD IP	0.520
				SAL CT-SAL IP	**0.000**
				SAL CT- PD CT	0.880
				SAL CT- PD IP	**0.000**
				SAL IP - PD CT	**0.000**
				SAL IP - PD IP	0.350
				PD CT - PD IP	**0.000**
697	Welch's ANOVA	75.368	0	Games-Howell	
				CTRL - SAL CT	**0.000**
				CTRL - SAL IP	**0.030**
				CTRL - PD CT	**0.000**
				CTRL - PD IP	**0.010**
				SAL CT-SAL IP	**0.000**
				SAL CT- PD CT	1.000
				SAL CT- PD IP	**0.000**
				SAL IP - PD CT	**0.000**
				SAL IP - PD IP	1.000
				PD CT - PD IP	**0.000**
702	Welch's ANOVA	14.748	0	Games-Howell	
				CTRL - SAL CT	0.640
				CTRL - SAL IP	**0.000**
				CTRL - PD CT	0.120

				CTRL - PD IP	**0.030**
				SAL CT-SAL IP	**0.000**
				SAL CT- PD CT	0.940
				SAL CT- PD IP	**0.010**
				SAL IP - PD CT	**0.000**
				SAL IP - PD IP	0.480
				PD CT - PD IP	**0.000**
704	Welch's ANOVA	42.376	0	Games-Howell	
				CTRL - SAL CT	**0.000**
				CTRL - SAL IP	**0.000**
				CTRL - PD CT	**0.000**
				CTRL - PD IP	**0.010**
				SAL CT-SAL IP	**0.000**
				SAL CT- PD CT	0.570
				SAL CT- PD IP	**0.000**
				SAL IP - PD CT	**0.000**
				SAL IP - PD IP	1.000
				PD CT - PD IP	**0.000**
705	ANOVA	59.005	0	Bonferroni	
				CTRL - SAL CT	**0.000**
				CTRL - SAL IP	1.000
				CTRL - PD CT	**0.000**
				CTRL - PD IP	1.000
				SAL CT-SAL IP	**0.000**
				SAL CT- PD CT	1.000
				SAL CT- PD IP	**0.000**
				SAL IP - PD CT	**0.000**
				SAL IP - PD IP	1.000
				PD CT - PD IP	**0.000**
717	ANOVA	72.988	0	Bonferroni	
				CTRL - SAL CT	**0.000**
				CTRL - SAL IP	1.000
				CTRL - PD CT	**0.000**
				CTRL - PD IP	1.000
				SAL CT-SAL IP	**0.000**
				SAL CT- PD CT	1.000
				SAL CT- PD IP	**0.000**
				SAL IP - PD CT	**0.000**
				SAL IP - PD IP	1.000
				PD CT - PD IP	**0.000**
724	ANOVA	46.840	0	Bonferroni	
				CTRL - SAL CT	**0.000**
				CTRL - SAL IP	1.000

				CTRL - PD CT	**0.000**
				CTRL - PD IP	0.760
				SAL CT-SAL IP	**0.000**
				SAL CT- PD CT	1.000
				SAL CT- PD IP	**0.000**
				SAL IP - PD CT	**0.000**
				SAL IP - PD IP	1.000
				PD CT - PD IP	**0.000**
750	Welch's ANOVA	154.948	0	Games-Howell	
				CTRL - SAL CT	**0.000**
				CTRL - SAL IP	**0.000**
				CTRL - PD CT	**0.000**
				CTRL - PD IP	**0.000**
				SAL CT-SAL IP	**0.000**
				SAL CT- PD CT	0.950
				SAL CT- PD IP	**0.000**
				SAL IP - PD CT	**0.000**
				SAL IP - PD IP	0.950
				PD CT - PD IP	**0.000**
801	ANOVA	84.962	0	Bonferroni	
				CTRL - SAL CT	**0.000**
				CTRL - SAL IP	1.000
				CTRL - PD CT	**0.000**
				CTRL - PD IP	0.100
				SAL CT-SAL IP	**0.000**
				SAL CT- PD CT	1.000
				SAL CT- PD IP	**0.000**
				SAL IP - PD CT	**0.000**
				SAL IP - PD IP	0.340
				PD CT - PD IP	**0.000**
809	ANOVA	87.803	0	Bonferroni	
				CTRL - SAL CT	**0.000**
				CTRL - SAL IP	0.070
				CTRL - PD CT	**0.000**
				CTRL - PD IP	**0.000**
				SAL CT-SAL IP	**0.000**
				SAL CT- PD CT	1.000
				SAL CT- PD IP	**0.000**
				SAL IP - PD CT	**0.000**
				SAL IP - PD IP	0.750
				PD CT - PD IP	**0.000**
831	ANOVA	58.816	0	Bonferroni	
				CTRL - SAL CT	**0.000**

				CTRL - SAL IP	1.000
				CTRL - PD CT	**0.000**
				CTRL - PD IP	0.110
				SAL CT-SAL IP	**0.000**
				SAL CT- PD CT	1.000
				SAL CT- PD IP	**0.000**
				SAL IP - PD CT	**0.000**
				SAL IP - PD IP	**0.030**
				PD CT - PD IP	**0.000**
836	ANOVA	39.625	0	Bonferroni	
				CTRL - SAL CT	**0.000**
				CTRL - SAL IP	0.470
				CTRL - PD CT	**0.000**
				CTRL - PD IP	1.000
				SAL CT-SAL IP	**0.000**
				SAL CT- PD CT	1.000
				SAL CT- PD IP	**0.000**
				SAL IP - PD CT	**0.000**
				SAL IP - PD IP	1.000
				PD CT - PD IP	**0.000**
845	ANOVA	48.062	0	Bonferroni	
				CTRL - SAL CT	**0.000**
				CTRL - SAL IP	**0.030**
				CTRL - PD CT	**0.000**
				CTRL - PD IP	1.000
				SAL CT-SAL IP	**0.000**
				SAL CT- PD CT	1.000
				SAL CT- PD IP	**0.000**
				SAL IP - PD CT	**0.000**
				SAL IP - PD IP	1.000
				PD CT - PD IP	**0.000**
890	Welch's ANOVA	27.527	0	Games-Howell	
				CTRL - SAL CT	**0.000**
				CTRL - SAL IP	**0.000**
				CTRL - PD CT	**0.000**
				CTRL - PD IP	0.130
				SAL CT-SAL IP	**0.000**
				SAL CT- PD CT	0.820
				SAL CT- PD IP	**0.000**
				SAL IP - PD CT	**0.000**
				SAL IP - PD IP	0.810
				PD CT - PD IP	**0.000**
900	ANOVA	46.757	0	Bonferroni	

				CTRL - SAL CT	**0.000**
				CTRL - SAL IP	1.000
				CTRL - PD CT	**0.000**
				CTRL - PD IP	1.000
				SAL CT-SAL IP	**0.000**
				SAL CT- PD CT	1.000
				SAL CT- PD IP	**0.000**
				SAL IP - PD CT	**0.000**
				SAL IP - PD IP	1.000
				PD CT - PD IP	**0.000**
930	ANOVA	39.186	0	Bonferroni	
				CTRL - SAL CT	**0.000**
				CTRL - SAL IP	1.000
				CTRL - PD CT	**0.000**
				CTRL - PD IP	1.000
				SAL CT-SAL IP	**0.000**
				SAL CT- PD CT	1.000
				SAL CT- PD IP	**0.000**
				SAL IP - PD CT	**0.000**
				SAL IP - PD IP	1.000
				PD CT - PD IP	**0.000**
945	Welch's ANOVA	47.045	0	Games-Howell	
				CTRL - SAL CT	**0.000**
				CTRL - SAL IP	0.440
				CTRL - PD CT	**0.000**
				CTRL - PD IP	0.240
				SAL CT-SAL IP	**0.000**
				SAL CT- PD CT	0.820
				SAL CT- PD IP	**0.000**
				SAL IP - PD CT	**0.000**
				SAL IP - PD IP	1.000
				PD CT - PD IP	**0.000**
1003	Welch's ANOVA	23.412	0	Games-Howell	
				CTRL - SAL CT	**0.000**
				CTRL - SAL IP	0.960
				CTRL - PD CT	**0.000**
				CTRL - PD IP	1.000
				SAL CT-SAL IP	**0.000**
				SAL CT- PD CT	**0.020**
				SAL CT- PD IP	**0.000**
				SAL IP - PD CT	**0.000**
				SAL IP - PD IP	0.950
				PD CT - PD IP	**0.010**

1006	Welch's ANOVA	50.704	0	Games-Howell	
				CTRL - SAL CT	**0.000**
				CTRL - SAL IP	0.470
				CTRL - PD CT	**0.000**
				CTRL - PD IP	1.000
				SAL CT-SAL IP	**0.000**
				SAL CT- PD CT	**0.000**
				SAL CT- PD IP	**0.000**
				SAL IP - PD CT	**0.000**
				SAL IP - PD IP	0.730
				PD CT - PD IP	**0.000**
1010	ANOVA	105.886	0	Bonferroni	
				CTRL - SAL CT	**0.000**
				CTRL - SAL IP	1.000
				CTRL - PD CT	**0.000**
				CTRL - PD IP	0.860
				SAL CT-SAL IP	**0.000**
				SAL CT- PD CT	0.520
				SAL CT- PD IP	**0.000**
				SAL IP - PD CT	**0.000**
				SAL IP - PD IP	0.670
				PD CT - PD IP	**0.000**
1016	ANOVA	100.612	0	Bonferroni	
				CTRL - SAL CT	**0.000**
				CTRL - SAL IP	1.000
				CTRL - PD CT	**0.000**
				CTRL - PD IP	0.270
				SAL CT-SAL IP	**0.000**
				SAL CT- PD CT	**0.040**
				SAL CT- PD IP	**0.000**
				SAL IP - PD CT	**0.000**
				SAL IP - PD IP	0.340
				PD CT - PD IP	**0.000**
1026	Welch's ANOVA	75.343	0	Games-Howell	
				CTRL - SAL CT	**0.000**
				CTRL - SAL IP	0.700
				CTRL - PD CT	**0.000**
				CTRL - PD IP	**0.000**
				SAL CT-SAL IP	**0.000**
				SAL CT- PD CT	**0.000**
				SAL CT- PD IP	**0.000**
				SAL IP - PD CT	**0.000**
				SAL IP - PD IP	0.060

				PD CT - PD IP	**0.000**
1044	Welch's ANOVA	86.803	0	Games-Howell	
				CTRL - SAL CT	**0.000**
				CTRL - SAL IP	0.140
				CTRL - PD CT	**0.000**
				CTRL - PD IP	**0.000**
				SAL CT-SAL IP	**0.000**
				SAL CT- PD CT	0.110
				SAL CT- PD IP	**0.000**
				SAL IP - PD CT	**0.000**
				SAL IP - PD IP	0.050
				PD CT - PD IP	**0.000**
1045	Welch's ANOVA	28.611	0	Games-Howell	
				CTRL - SAL CT	0.110
				CTRL - SAL IP	0.270
				CTRL - PD CT	**0.000**
				CTRL - PD IP	**0.000**
				SAL CT-SAL IP	0.700
				SAL CT- PD CT	**0.000**
				SAL CT- PD IP	1.000
				SAL IP - PD CT	**0.000**
				SAL IP - PD IP	0.060
				PD CT - PD IP	**0.000**
1134	Welch's ANOVA	7.461	0	Games-Howell	
				CTRL - SAL CT	**0.000**
				CTRL - SAL IP	0.360
				CTRL - PD CT	**0.000**
				CTRL - PD IP	0.790
				SAL CT-SAL IP	**0.000**
				SAL CT- PD CT	0.840
				SAL CT- PD IP	**0.000**
				SAL IP - PD CT	**0.000**
				SAL IP - PD IP	0.990
				PD CT - PD IP	**0.000**
1157	Welch's ANOVA	17.616	0	Games-Howell	
				CTRL - SAL CT	**0.010**
				CTRL - SAL IP	0.770
				CTRL - PD CT	**0.000**
				CTRL - PD IP	**0.040**
				SAL CT-SAL IP	**0.020**
				SAL CT- PD CT	**0.000**
				SAL CT- PD IP	**0.050**
				SAL IP - PD CT	**0.000**

				SAL IP - PD IP	0.750
				PD CT - PD IP	**0.000**
1306	Welch's ANOVA	188.067	0	Games-Howell	
				CTRL - SAL CT	**0.000**
				CTRL - SAL IP	**0.000**
				CTRL - PD CT	**0.000**
				CTRL - PD IP	**0.000**
				SAL CT-SAL IP	**0.000**
				SAL CT- PD CT	**0.000**
				SAL CT- PD IP	**0.000**
				SAL IP - PD CT	**0.000**
				SAL IP - PD IP	0.070
				PD CT - PD IP	**0.000**
1320	Welch's ANOVA	235.217	0	Games-Howell	
				CTRL - SAL CT	**0.000**
				CTRL - SAL IP	0.350
				CTRL - PD CT	**0.000**
				CTRL - PD IP	**0.000**
				SAL CT-SAL IP	**0.000**
				SAL CT- PD CT	**0.000**
				SAL CT- PD IP	**0.000**
				SAL IP - PD CT	**0.000**
				SAL IP - PD IP	0.670
				PD CT - PD IP	**0.000**
1342	Welch's ANOVA	248.611	0	Games-Howell	
				CTRL - SAL CT	**0.000**
				CTRL - SAL IP	**0.050**
				CTRL - PD CT	**0.000**
				CTRL - PD IP	0.840
				SAL CT-SAL IP	**0.000**
				SAL CT- PD CT	**0.000**
				SAL CT- PD IP	**0.000**
				SAL IP - PD CT	**0.000**
				SAL IP - PD IP	**0.020**
				PD CT - PD IP	**0.000**
1360	Welch's ANOVA	576.371	0	Games-Howell	
				CTRL - SAL CT	**0.000**
				CTRL - SAL IP	**0.000**
				CTRL - PD CT	**0.000**
				CTRL - PD IP	0.740
				SAL CT-SAL IP	**0.000**
				SAL CT- PD CT	**0.000**
				SAL CT- PD IP	**0.000**

				SAL IP - PD CT	**0.000**
				SAL IP - PD IP	**0.030**
				PD CT - PD IP	**0.000**
1390	Welch's ANOVA	192.953	0	Games-Howell	
				CTRL - SAL CT	**0.000**
				CTRL - SAL IP	**0.080**
				CTRL - PD CT	**0.000**
				CTRL - PD IP	0.670
				SAL CT-SAL IP	**0.000**
				SAL CT- PD CT	0.880
				SAL CT- PD IP	**0.000**
				SAL IP - PD CT	**0.000**
				SAL IP - PD IP	0.430
				PD CT - PD IP	**0.000**
1400	ANOVA	419.644	0	Bonferroni	
				CTRL - SAL CT	**0.000**
				CTRL - SAL IP	1.000
				CTRL - PD CT	**0.000**
				CTRL - PD IP	1.000
				SAL CT-SAL IP	**0.000**
				SAL CT- PD CT	1.000
				SAL CT- PD IP	**0.000**
				SAL IP - PD CT	**0.000**
				SAL IP - PD IP	1.000
				PD CT - PD IP	**0.000**
1405	ANOVA	214.107	0	Bonferroni	
				CTRL - SAL CT	**0.000**
				CTRL - SAL IP	0.960
				CTRL - PD CT	**0.000**
				CTRL - PD IP	**0.010**
				SAL CT-SAL IP	**0.000**
				SAL CT- PD CT	**0.000**
				SAL CT- PD IP	**0.000**
				SAL IP - PD CT	**0.000**
				SAL IP - PD IP	1.000
				PD CT - PD IP	**0.000**
1417	Welch's ANOVA	99.833	0	Games-Howell	
				CTRL - SAL CT	**0.000**
				CTRL - SAL IP	0.940
				CTRL - PD CT	**0.000**
				CTRL - PD IP	0.420
				SAL CT-SAL IP	**0.000**
				SAL CT- PD CT	**0.010**

				SAL CT- PD IP	**0.000**
				SAL IP - PD CT	**0.000**
				SAL IP - PD IP	0.670
				PD CT - PD IP	**0.000**
1419	Welch's ANOVA	74.660	0	Games-Howell	
				CTRL - SAL CT	**0.000**
				CTRL - SAL IP	0.910
				CTRL - PD CT	**0.000**
				CTRL - PD IP	0.150
				SAL CT-SAL IP	**0.000**
				SAL CT- PD CT	0.300
				SAL CT- PD IP	**0.000**
				SAL IP - PD CT	**0.000**
				SAL IP - PD IP	0.310
				PD CT - PD IP	**0.000**
1420	Welch's ANOVA	39.734	0	Games-Howell	
				CTRL - SAL CT	**0.000**
				CTRL - SAL IP	0.950
				CTRL - PD CT	**0.000**
				CTRL - PD IP	0.190
				SAL CT-SAL IP	**0.000**
				SAL CT- PD CT	**0.000**
				SAL CT- PD IP	0.800
				SAL IP - PD CT	**0.000**
				SAL IP - PD IP	0.350
				PD CT - PD IP	**0.000**
1428	Welch's ANOVA	103.486	0	Games-Howell	
				CTRL - SAL CT	**0.000**
				CTRL - SAL IP	**0.050**
				CTRL - PD CT	**0.000**
				CTRL - PD IP	0.990
				SAL CT-SAL IP	**0.000**
				SAL CT- PD CT	**0.000**
				SAL CT- PD IP	**0.000**
				SAL IP - PD CT	**0.000**
				SAL IP - PD IP	0.460
				PD CT - PD IP	**0.000**
1439	Welch's ANOVA	13.355	0	Games-Howell	
				CTRL - SAL CT	**0.000**
				CTRL - SAL IP	0.170
				CTRL - PD CT	**0.000**
				CTRL - PD IP	**0.050**
				SAL CT-SAL IP	**0.010**

				SAL CT- PD CT	**0.000**
				SAL CT- PD IP	**0.030**
				SAL IP - PD CT	0.880
				SAL IP - PD IP	0.990
				PD CT - PD IP	1.000
1445	Welch's ANOVA	11.772	0	Games-Howell	
				CTRL - SAL CT	**0.000**
				CTRL - SAL IP	0.200
				CTRL - PD CT	**0.000**
				CTRL - PD IP	0.100
				SAL CT-SAL IP	**0.040**
				SAL CT- PD CT	0.720
				SAL CT- PD IP	0.080
				SAL IP - PD CT	0.320
				SAL IP - PD IP	1.000
				PD CT - PD IP	0.490
1450	Welch's ANOVA	11.633	0	Games-Howell	
				CTRL - SAL CT	**0.000**
				CTRL - SAL IP	0.140
				CTRL - PD CT	**0.000**
				CTRL - PD IP	**0.020**
				SAL CT-SAL IP	0.350
				SAL CT- PD CT	0.720
				SAL CT- PD IP	0.800
				SAL IP - PD CT	**0.050**
				SAL IP - PD IP	0.970
				PD CT - PD IP	0.240
1454	Welch's ANOVA	14.664	0	Games-Howell	
				CTRL - SAL CT	**0.000**
				CTRL - SAL IP	0.060
				CTRL - PD CT	**0.000**
				CTRL - PD IP	**0.010**
				SAL CT-SAL IP	0.150
				SAL CT- PD CT	0.830
				SAL CT- PD IP	0.730
				SAL IP - PD CT	**0.010**
				SAL IP - PD IP	0.930
				PD CT - PD IP	0.240
1460	Welch's ANOVA	203.574	0	Games-Howell	
				CTRL - SAL CT	**0.000**
				CTRL - SAL IP	0.070
				CTRL - PD CT	**0.000**
				CTRL - PD IP	**0.000**

				SAL CT-SAL IP	**0.000**
				SAL CT- PD CT	**0.000**
				SAL CT- PD IP	**0.000**
				SAL IP - PD CT	**0.020**
				SAL IP - PD IP	0.910
				PD CT - PD IP	0.210
1520	ANOVA	216.959	0	Bonferroni	
				CTRL - SAL CT	**0.000**
				CTRL - SAL IP	1.000
				CTRL - PD CT	**0.000**
				CTRL - PD IP	1.000
				SAL CT-SAL IP	**0.000**
				SAL CT- PD CT	1.000
				SAL CT- PD IP	**0.000**
				SAL IP - PD CT	**0.000**
				SAL IP - PD IP	1.000
				PD CT - PD IP	**0.000**
1534	ANOVA	152.575	0	Bonferroni	
				CTRL - SAL CT	**0.000**
				CTRL - SAL IP	1.000
				CTRL - PD CT	**0.000**
				CTRL - PD IP	1.000
				SAL CT-SAL IP	**0.000**
				SAL CT- PD CT	**0.000**
				SAL CT- PD IP	**0.000**
				SAL IP - PD CT	**0.000**
				SAL IP - PD IP	0.970
				PD CT - PD IP	**0.000**
1580	ANOVA	108.359	0	Bonferroni	
				CTRL - SAL CT	**0.000**
				CTRL - SAL IP	1.000
				CTRL - PD CT	**0.000**
				CTRL - PD IP	1.000
				SAL CT-SAL IP	**0.000**
				SAL CT- PD CT	**0.000**
				SAL CT- PD IP	**0.000**
				SAL IP - PD CT	**0.000**
				SAL IP - PD IP	1.000
				PD CT - PD IP	**0.000**
1612	Welch's ANOVA	183.406	0	Games-Howell	
				CTRL - SAL CT	**0.000**
				CTRL - SAL IP	**0.010**
				CTRL - PD CT	**0.000**

				CTRL - PD IP	0.310
				SAL CT-SAL IP	**0.000**
				SAL CT- PD CT	0.060
				SAL CT- PD IP	**0.000**
				SAL IP - PD CT	**0.000**
				SAL IP - PD IP	0.310
				PD CT - PD IP	**0.000**
1620	Welch's ANOVA	148.572	0	Games-Howell	
				CTRL - SAL CT	**0.000**
				CTRL - SAL IP	**0.010**
				CTRL - PD CT	**0.000**
				CTRL - PD IP	**0.010**
				SAL CT-SAL IP	**0.000**
				SAL CT- PD CT	**0.050**
				SAL CT- PD IP	**0.000**
				SAL IP - PD CT	**0.000**
				SAL IP - PD IP	0.980
				PD CT - PD IP	**0.000**
1630	ANOVA	110.220	0	Bonferroni	
				CTRL - SAL CT	**0.000**
				CTRL - SAL IP	**0.010**
				CTRL - PD CT	**0.000**
				CTRL - PD IP	**0.010**
				SAL CT-SAL IP	**0.000**
				SAL CT- PD CT	1.000
				SAL CT- PD IP	**0.000**
				SAL IP - PD CT	**0.000**
				SAL IP - PD IP	1.000
				PD CT - PD IP	**0.000**
1632	ANOVA	96.668	0	Bonferroni	
				CTRL - SAL CT	**0.000**
				CTRL - SAL IP	**0.000**
				CTRL - PD CT	**0.000**
				CTRL - PD IP	**0.010**
				SAL CT-SAL IP	**0.000**
				SAL CT- PD CT	1.000
				SAL CT- PD IP	**0.000**
				SAL IP - PD CT	**0.000**
				SAL IP - PD IP	1.000
				PD CT - PD IP	**0.000**
1635	ANOVA	102.511	0	Bonferroni	
				CTRL - SAL CT	**0.000**
				CTRL - SAL IP	**0.000**

				CTRL - PD CT	**0.000**
				CTRL - PD IP	**0.020**
				SAL CT-SAL IP	**0.000**
				SAL CT- PD CT	1.000
				SAL CT- PD IP	**0.000**
				SAL IP - PD CT	**0.000**
				SAL IP - PD IP	1.000
				PD CT - PD IP	**0.000**
1637	ANOVA	81.865	0	Bonferroni	
				CTRL - SAL CT	**0.000**
				CTRL - SAL IP	**0.010**
				CTRL - PD CT	**0.000**
				CTRL - PD IP	0.130
				SAL CT-SAL IP	**0.000**
				SAL CT- PD CT	1.000
				SAL CT- PD IP	**0.000**
				SAL IP - PD CT	**0.000**
				SAL IP - PD IP	1.000
				PD CT - PD IP	**0.000**
1640	Welch's ANOVA	70.363	0	Games-Howell	
				CTRL - SAL CT	**0.000**
				CTRL - SAL IP	**0.010**
				CTRL - PD CT	**0.000**
				CTRL - PD IP	0.170
				SAL CT-SAL IP	**0.000**
				SAL CT- PD CT	0.110
				SAL CT- PD IP	**0.000**
				SAL IP - PD CT	**0.000**
				SAL IP - PD IP	0.920
				PD CT - PD IP	**0.000**
1645	Welch's ANOVA	36.317	0	Games-Howell	
				CTRL - SAL CT	**0.000**
				CTRL - SAL IP	**0.010**
				CTRL - PD CT	**0.000**
				CTRL - PD IP	**0.000**
				SAL CT-SAL IP	**0.010**
				SAL CT- PD CT	**0.010**
				SAL CT- PD IP	**0.010**
				SAL IP - PD CT	**0.000**
				SAL IP - PD IP	1.000
				PD CT - PD IP	**0.000**
1650	Welch's ANOVA	27.108	0	Games-Howell	
				CTRL - SAL CT	**0.000**

				CTRL - SAL IP	**0.010**
				CTRL - PD CT	**0.000**
				CTRL - PD IP	**0.000**
				SAL CT-SAL IP	0.100
				SAL CT- PD CT	**0.010**
				SAL CT- PD IP	0.460
				SAL IP - PD CT	**0.000**
				SAL IP - PD IP	0.860
				PD CT - PD IP	**0.000**
1655	ANOVA	32.012	0	Bonferroni	
				CTRL - SAL CT	**0.000**
				CTRL - SAL IP	**0.000**
				CTRL - PD CT	**0.000**
				CTRL - PD IP	**0.000**
				SAL CT-SAL IP	**0.000**
				SAL CT- PD CT	1.000
				SAL CT- PD IP	**0.000**
				SAL IP - PD CT	**0.000**
				SAL IP - PD IP	1.000
				PD CT - PD IP	**0.010**
1665	ANOVA	20.204	0	Bonferroni	
				CTRL - SAL CT	**0.000**
				CTRL - SAL IP	1.000
				CTRL - PD CT	**0.000**
				CTRL - PD IP	**0.000**
				SAL CT-SAL IP	**0.000**
				SAL CT- PD CT	1.000
				SAL CT- PD IP	**0.020**
				SAL IP - PD CT	**0.000**
				SAL IP - PD IP	0.570
				PD CT - PD IP	0.080
1666	ANOVA	18.579	0	Bonferroni	
				CTRL - SAL CT	**0.000**
				CTRL - SAL IP	1.000
				CTRL - PD CT	**0.000**
				CTRL - PD IP	**0.000**
				SAL CT-SAL IP	**0.000**
				SAL CT- PD CT	1.000
				SAL CT- PD IP	**0.030**
				SAL IP - PD CT	**0.000**
				SAL IP - PD IP	0.750
				PD CT - PD IP	**0.050**
1670	Welch's ANOVA	76.325	0	Games-Howell	

				CTRL - SAL CT	**0.000**
				CTRL - SAL IP	1.000
				CTRL - PD CT	**0.000**
				CTRL - PD IP	**0.030**
				SAL CT-SAL IP	**0.000**
				SAL CT- PD CT	**0.000**
				SAL CT- PD IP	**0.000**
				SAL IP - PD CT	**0.000**
				SAL IP - PD IP	0.100
				PD CT - PD IP	0.150
1680	ANOVA	117.469	0	Bonferroni	
				CTRL - SAL CT	**0.000**
				CTRL - SAL IP	1.000
				CTRL - PD CT	**0.000**
				CTRL - PD IP	0.990
				SAL CT-SAL IP	**0.000**
				SAL CT- PD CT	**0.000**
				SAL CT- PD IP	**0.000**
				SAL IP - PD CT	**0.000**
				SAL IP - PD IP	1.000
				PD CT - PD IP	**0.000**
1690	ANOVA	167.391	0	Bonferroni	
				CTRL - SAL CT	**0.000**
				CTRL - SAL IP	1.000
				CTRL - PD CT	**0.000**
				CTRL - PD IP	1.000
				SAL CT-SAL IP	**0.000**
				SAL CT- PD CT	**0.000**
				SAL CT- PD IP	**0.000**
				SAL IP - PD CT	**0.000**
				SAL IP - PD IP	1.000
				PD CT - PD IP	**0.000**
1697	ANOVA	270.198	0	Bonferroni	
				CTRL - SAL CT	**0.000**
				CTRL - SAL IP	1.000
				CTRL - PD CT	**0.000**
				CTRL - PD IP	1.000
				SAL CT-SAL IP	**0.000**
				SAL CT- PD CT	1.000
				SAL CT- PD IP	**0.000**
				SAL IP - PD CT	**0.000**
				SAL IP - PD IP	1.000
				PD CT - PD IP	**0.000**

1734	ANOVA	350.765	0	Bonferroni	
				CTRL - SAL CT	**0.000**
				CTRL - SAL IP	1.000
				CTRL - PD CT	**0.000**
				CTRL - PD IP	1.000
				SAL CT-SAL IP	**0.000**
				SAL CT- PD CT	0.840
				SAL CT- PD IP	**0.000**
				SAL IP - PD CT	**0.000**
				SAL IP - PD IP	1.000
				PD CT - PD IP	**0.000**
2255	ANOVA	558.757	0	Bonferroni	
				CTRL - SAL CT	**0.000**
				CTRL - SAL IP	**0.000**
				CTRL - PD CT	**0.000**
				CTRL - PD IP	1.000
				SAL CT-SAL IP	**0.000**
				SAL CT- PD CT	0.390
				SAL CT- PD IP	**0.000**
				SAL IP - PD CT	**0.000**
				SAL IP - PD IP	0.160
				PD CT - PD IP	**0.000**

Table S7. *Statistical analysis of the DNP Raman peaks described in the literature. Either one-way ANOVA or Welch's ANOVA was performed, followed by post hoc test Games-Howell or Bonferroni, with # p<0.05 and ##p<0.001. Sample distribution was initially tested for normality using the Shapiro-Wilk test, and Levene's for testing the homogeneity of variances.*

Wavelength (cm^{-1})	Type	F	p	*Post hoc* test	p
848	ANOVA	6.106	0	Bonferroni	
				CTRL - SAL CT	1.000
				CTRL - SAL IP	0.377
				CTRL - PD CT	0.277
				CTRL - PD IP	1.000
				SAL CT - SAL IP	1.000
				SAL CT - PD CT	**0.006**
				SAL CT - PD IP	**0.023**
				SAL IP - PD CT	**0.002**
				SAL IP - PD IP	**0.006**
				PD CT - PD IP	1.000
1139	ANOVA	16.068	0	Bonferroni	
				CTRL - SAL CT	**0.000**
				CTRL - SAL IP	**0.000**
				CTRL - PD CT	**0.002**
				CTRL - PD IP	**0.000**
				SAL CT - SAL IP	1.000
				SAL CT - PD CT	1.000
				SAL CT - PD IP	0.159
				SAL IP - PD CT	1.000
				SAL IP - PD IP	0.455
				PD CT - PD IP	0.112
1332	ANOVA	8.339	0	Bonferroni	
				CTRL - SAL CT	**0.001**
				CTRL - SAL IP	**0.012**
				CTRL - PD CT	**0.038**
				CTRL - PD IP	**0.000**
				SAL CT - SAL IP	1.000
				SAL CT - PD CT	1.000
				SAL CT - PD IP	0.874
				SAL IP - PD CT	1.000
				SAL IP - PD IP	0.269
				PD CT - PD IP	0.891
1340	ANOVA	8.562	0	Bonferroni	
				CTRL - SAL CT	**0.000**
				CTRL - SAL IP	**0.001**
				CTRL - PD CT	**0.070**
				CTRL - PD IP	**0.000**

				SAL CT - SAL IP	1.000
				SAL CT - PD CT	1.000
				SAL CT - PD IP	1,000
				SAL IP - PD CT	1.000
				SAL IP - PD IP	1.000
				PD CT - PD IP	1.000
1350	ANOVA	9.475	0	Bonferroni	
				CTRL - SAL CT	0.273
				CTRL - SAL IP	0.949
				CTRL - PD CT	**0.011**
				CTRL - PD IP	**0.000**
				SAL CT - SAL IP	1.000
				SAL CT - PD CT	1.000
				SAL CT - PD IP	**0.004**
				SAL IP - PD CT	0.636
				SAL IP - PD IP	**0.001**
				PD CT - PD IP	1.000
1600	Welch ANOVA	20.566	0	Games-Howell	
				CTRL - SAL CT	**0.007**
				CTRL - SAL IP	**0.001**
				CTRL - PD CT	**0.006**
				CTRL - PD IP	**0.000**
				SAL CT - SAL IP	0.912
				SAL CT - PD CT	0.414
				SAL CT - PD IP	**0.000**
				SAL IP - PD CT	0.775
				SAL IP - PD IP	**0.001**
				PD CT - PD IP	**0.029**
